# Comparison of biomarkers of exposure among US adult smokers, users of electronic nicotine delivery systems, dual users and nonusers, 2018–2019

**DOI:** 10.1038/s41598-023-34427-x

**Published:** 2023-05-05

**Authors:** Nathan M. Holt, Saul Shiffman, Ryan A. Black, Nicholas I. Goldenson, Mark A. Sembower, Michael J. Oldham

**Affiliations:** 1 Juul Labs, Inc., Washington, DC USA; 2 Pinney Associates, Inc., Pittsburgh, PA USA

**Keywords:** Biomarkers, Risk factors

## Abstract

The harm caused by cigarette smoking is overwhelmingly due to byproducts of tobacco combustion. Electronic Nicotine Delivery Systems (ENDS) provide nicotine to users without combustion, and may support tobacco harm reduction among cigarette smokers who would not otherwise quit in the near term. Analyses of Wave 5 of the Population Assessment of Tobacco and Health (PATH) Study compared biomarkers of exposure (BOE) levels for nicotine, 3 metals, 2 tobacco-specific nitrosamines and 14 smoking-related volatile organic compounds in 151 exclusive ENDS users, 1341 exclusive cigarette smokers, 115 dual users (cigarettes and ENDS), and 1846 past 30-day nonusers of tobacco, adjusting for demographics. Nicotine exposure in ENDS users and dual users did not significantly differ from smokers. Among ENDS users, 16 of 18 other BOEs were significantly lower than smokers’; 9 BOEs were not significantly different from nonusers. Among dual users smoking < 10 cigarettes/day, 15 of 18 non-nicotine BOEs were significantly lower than smokers’, whereas in dual users smoking ≥ 10 cigarettes per day none of the BOEs significantly differed from smokers’. In this representative sample of US adults, exclusive use of ENDS (vs. cigarette smoking) was associated with much lower exposures to many harmful chemicals associated with smoking-related disease. BOE levels in dual users were directly related to their cigarette consumption. These BOE data provide further evidence that ENDS expose users to substantially lower levels of toxicants than combustible cigarettes, confirming their potential for harm reduction.

## Introduction

Cigarette smoking is responsible for more than seven million deaths annually worldwide^1–3^. The harm caused by cigarette smoking is primarily due to exposure to byproducts of tobacco combustion^4^: cigarette smoke contains more than 7000 harmful chemicals including nearly 70 carcinogens. The US Food and Drug Administration has identified harmful and potentially harmful constituents (HPHCs) in cigarette smoke and in other tobacco products that are known to cause cancer and cardiovascular and respiratory diseases^5,6^. The risk and severity of many smoking-induced diseases are directly related to the level of exposure to the HPHCs in cigarette smoke^5^; hence reductions in exposure to HPHCs are expected to reduce risk for these diseases and many other adverse health effects^7,8^.

Many HPHCs are not directly measurable in the bodies of smokers; however, exposures can be assessed and quantified by measuring biomarkers of exposure (BOEs)—metabolites of the HPHC that are detectable in the urine and bloodstream^7,8^. BOEs provide a measure of the actual human HPHC absorption associated with tobacco use, and thus can help quantify the potential health risks of tobacco products^7,8^.

In contrast to cigarettes, electronic nicotine delivery systems (ENDS) deliver nicotine without combusting tobacco^9^, and thus do not expose users to combustion byproducts such as carbon monoxide (CO) and polycyclic aromatic hydrocarbons^9–11^. Clinical trials demonstrate that smokers who switch to exclusive ENDS use experience significant reductions in tobacco specific nitrosamines (TSNAs), such as NNAL—a biomarker for NNK, and known carcinogen^12^.

However, there is a need to establish whether, like cigarettes, ENDS expose users to volatile organic compounds (VOCs)—HPHCs that exhibit dose–response relationships with cancer and cardiovascular disease risk^13^. For example, acrolein (whose exposure can be assessed using 3-hydroxypropyl-mercapturic acid [3-HPMA]) is a respiratory irritant that is also associated with increased risk of cardiovascular disease^14^, and 1,3-butadiene (whose exposure can be assessed using monohydroxylbutenyl-mercapturic acid [MHBMA]) is a known human carcinogen and is also associated with respiratory and reproductive toxicity^15–17^. Additionally, some studies suggest metals can leech from heating coils into ENDS aerosols^18,19^, potentially causing adverse health effects given their high level of toxicity and carcinogenic effects^20,21^.

Several recent analyses have utilized BOE data from the Population Assessment of Tobacco and Health (PATH) Study, a longitudinal cohort study of tobacco use among a nationally representative sample of US adults, to assess variation in HPHC exposure. Cross-sectional analyses of PATH Wave 1 (2013–2014) demonstrated that exclusive ENDS users have lower levels of many of the measured BOEs compared to cigarette smokers and dual users^22,23^, and that among dual users, it is cigarette smoking, and not ENDS use, that is the primary driver of HPHC exposures^24–26^. Consistent with these findings, two longitudinal studies using data from PATH Wave 1 (2013–2014) to Wave 2 (2014–2015) found that smokers who switch from cigarette smoking to exclusive ENDS use experienced substantial decreases in BOEs, and smokers who transition to dual use and reduce their cigarette consumption by at least 50% also experience significant reductions in BOEs^27,28^.

Although these studies offer valuable insights into HPHC exposures among ENDS users, the findings are limited to early-generation ENDS products marketed when the data was collected between 2013–2015. ENDS products have rapidly evolved since 2015, with open system modular devices and fourth-generation pod-based nicotine-salt ENDS products becoming increasingly prevalent^29–31^. These later-generation products deliver nicotine more efficiently than earlier-generation products^32,33^, and some implement better control of the temperature to which the e-liquid is heated, a major factor in production of toxic compounds^34^; hence there is a need to assess differences in BOEs among ENDS users in more recently-collected data.

The primary aim of the current study was to assess differences in levels of BOEs to TSNAs, VOCs, metals and nicotine, comparing: (i) exclusive ENDS users; (ii) exclusive smokers; (iii) dual users; and (iv) past 30-day tobacco non-users using data from Wave 5 (2018–2019) of the PATH Study. Further, given heterogeneity in cigarette smoking behaviors among dual users^26,35^, exposures among dual users were also evaluated by their level of cigarette consumption.

## Materials and methods

### Study design

The PATH Study is a nationally-representative, longitudinal cohort study of adults and youth in the US^36^. Recruitment used a stratified address-based, area-probability sampling design; survey weights are used to produce national estimates^36^. The study was conducted by Westat under a contract with the Food and Drug Administration (FDA) Center for Tobacco Products (CTP), National Institute on Drug Abuse (NIDA) and National Institutes of Health (NIH). Westat’s Institutional Review Board (IRB) approved the study design and data collection protocol^36^. All participants in the PATH Study provided informed consent^36^. This secondary analysis of deidentified PATH Study data was reviewed by Advarra IRB Number IRB00000971 and deemed exempt on the basis of 45 CFR 46.104(d)(4). Use of these data was approved by the Inter-university Consortium for Political and Social Research (ICPSR), which reviewed analytic results and cleared these results for public dissemination on the basis that they did not present a risk of identifying individual participants. All procedures were performed in accordance with relevant guidelines.

The current analyses used data from Wave 5 (December 2018 to November 2019) of the PATH Study Biomarker Restricted-Use Files (BRUF)^37^ and Restricted-Use Files (RUF)^38^. Urine specimens were analyzed by the Centers for Disease Control and Prevention (CDC), National Center of Environmental Health. Because they contain more granular data which may pose a risk to respondent identification, the PATH Study BRUF and PATH Study RUF are only available to researchers that apply for access and complete a data use agreement, which includes review of statistical outputs before any results can be published. At this time, PATH Study biomarker data are available only in restricted-use files. Additional information is provided by^36^ and at 10.3886/ICPSR36840.v18 and 10.3886/ICPSR36231.v31.

### Participants

The analytic sample for the current manuscript is comprised of PATH Study Wave 5 adult respondents who were at least 21 years old at time of interview, provided urine samples and also had (i) valid data on sex and race/ethnicity; (ii) body mass index (BMI) between 18–40; (iii) urinary creatinine in the range 10–370 mg/dL; and (iv) valid BOE data on one or more analyte (including imputed data below the limit of detection [LOD]). To avoid confounding of BOEs by other exposures, participants were excluded if they reported past 30-day use of other tobacco, nicotine, or marijuana products (traditional or filtered cigars, cigarillos, pipe tobacco, hookah, smokeless tobacco, snus, nicotine replacement products, marijuana and hashish) at interview or past 3-day use at urine collection (Table S1). Further, respondents were excluded if they reported past 3-day product use behaviors at the time of urine collection inconsistent with their prior interview reports (e.g., reported no past-30-day smoking at interview but reported smoking in the past 3 days at urine collection). Smokers were excluded if their reported average daily cigarette consumption (i.e., cigarettes per day) was missing, zero, or greater than 100 (an unrealistically high number).

#### Tobacco product use groups

Participants were initially classified into one of four groups on the basis of past 30-day ENDS use and past 30-day cigarette smoking: (i) exclusive ENDS users (“ENDS Users”; past-30-day ENDS use and no past-30-day smoking); (ii) exclusive cigarette smokers (“Smokers”; past-30-day cigarette smoking, no past-30-day ENDS use); (iii) dual users (“Dual Users”; cigarette smoking and ENDS use in the past 30 days); and (iv) past 30-day tobacco product nonusers (“Nonusers”; neither smoking cigarettes nor using ENDS in the past 30 days). Preliminary analyses showed BOE levels were more heterogeneous in Dual Users than in other tobacco use groups (Figs. S1, S2, S3, and S4). Therefore, for the primary analyses reported here Dual Users were partitioned into two subgroups by the median daily cigarette consumption: those smoking < 10 cigarettes/day vs. those smoking ≥ 10 cigarettes/day (Table S2).

### Measures

#### BOE outcomes

In Version 18 of the PATH Study Biomarker Restricted-Use Files, available Wave 5 urine panels include BOE data for nicotine, metals, TSNAs, and VOCs. All 14 available VOC markers, two available TSNAs, and three available metals, as well as two aggregate measures of nicotine and its metabolites (total nicotine equivalents-2 [TNE2] and total nicotine equivalents-6 [TNE6]) were analyzed (Table 1). Table 1 displays the BOEs, noting their chemical name, the name of the parent compound, and the disease-related toxicities with which they are associated^6^. Altogether, 15 BOEs were considered to be relevant to cancer, 9 to respiratory disease, 8 to reproductive or developmental problems, and 4 to cardiovascular disease, with most BOEs relevant to multiple disease classes.

**Table 1:** Toxicological significance of PATH Study volatile organic compound (VOC), metal, tobacco-specific nitrosamine (TSNA), and nicotine biomarkers.

Toxicant	Chemical class	Toxicant type^1,2,3^	Measured urinary biomarker	Abbreviation code
Acrolein	VOC	CT, RT	*N*-Acetyl-S-(2-carboxyethyl)-l-cysteine	CEMA
			*N*-Acetyl-S-(3-hydroxypropyl)-l-cysteine	HPMA
Acrylamide	VOC	CA	*N*-Acetyl-S-(2-carbamoylethyl)-l-cysteine	AAMA
Acrylonitrile	VOC	CA, RT	*N*-Acetyl-S-(2-cyanoethyl)-l-cysteine	CYMA
Acrylonitrile, ethylene oxide, vinyl chloride	VOC	CA, RDT, RT	*N*-Acetyl-S-(2-hydroxyethyl)-l-cysteine	HEMA
Benzene	VOC	CA, CT, RDT	*N*-Acetyl-S-(phenyl)-l-cysteine	PMA
1,3-Butadiene	VOC	CA, RT, RDT	*N*-Acetyl-S-(4-hydroxy-2-buten-1-yl)-l-cysteine	MHB3
Crotonaldehyde	VOC	CA	*N*-Acetyl-S-(3-hydroxypropyl-1-methyl)-l-cysteine	HPMM
*N*,*N*-Dimethylforamide^2^, methyl isocyanate	VOC	RDT, RT	*N*-Acetyl-S-(*N*-methylcarbamoyl)-l-cysteine	AMCA
Ethylbenzene, styrene	VOC	CA	Phenylglyoxylic acid	PHGA
Isoprene	VOC	CA	*N*-Acetyl-S-(4-hydroxy-2-methyl-2-buten-1-yl)-l-cysteine	IMP3
Propylene oxide	VOC	CA, RT	*N*-Acetyl-S-(2-hydroxypropyl)-l-cysteine	HPM2
Styrene	VOC	CA	Mandelic acid	MADA
Xylene^3^	VOC	RDT	3-Methylhippuric acid + 4-Methylhippuric acid	34MH
4-(Methylnitrosamino)-1-(3-pyridyl)-1-butanone (NNK)	TSNA	CA	4-(Methylnitrosamino)-1-(3-pyridyl)-1-butanol	NNAL
*N*′-Nitrosonornicotine	TSNA	CA	*N*′-Nitrosonornicotine	NNN
Cadmium	Metal	CA, RDT, RT	Cadmium	UCD
Lead	Metal	CA, CT, RDT	Lead	UPB
Uranium^4^	Metal	CA, RT	Uranium	UUR
Nicotine	Nicotine	AD, RDT	Total Nicotine Equivalents-2	TNE2
Nicotine	Nicotine	AD, RDT	Total Nicotine Equivalents-6	TNE6

Toxicant, Measured Urinary Biomarker, and Abbreviation Code are drawn from PATH Study Biomarker Restricted Use Files Urinary Volatile Organic Compound Metabolites (VOCM) Laboratory Panel Documentation; Urinary Metals (Metals) Laboratory Panel Documentation; Urinary Tobacco-Specific Nitrosamines (TSNA) Laboratory Panel Documentation; and ICPSR Codebook for Wave 5: Urine Panel—Wave 1 Biomarker Core—Urinary Nicotine Metabolites (Cotinine and Hydroxycotinine) (UNICM). *AD* Addictive, *CA* Carcinogen, *CT* Cardiovascular toxicant, *RDT* Reproductive or developmental toxicant, *RT* Respiratory toxicant. ^1^According to the U.S. Food and Drug Administration^6^. ^2^According to U.S. Environmental Protection Agency, also liver toxicant; Hazard Summary, 2000. ^3^According to U.S. Agency for Toxic Substances and Disease Registry, also liver, neurological, and renal toxicant; Toxic Substance Portal last visited on August 5, 2022. ^4^Uranium-235 or Uranium-238.

### Data analysis

Urinary biomarker measurements were adjusted for urinary creatinine by expressing the concentration of the BOE as a ratio to the concentration of creatinine. This is an often-used adjustment for dilution of the spot urine samples by water^27^. Measurements below the LOD were imputed with the value LOD/$\sqrt {2}$ and then adjusted for urinary creatinine (10.3886/ICPSR36840.userguide). (Thus, levels are never considered to be zero.) Weighted geometric means and 95% confidence intervals were computed for each creatinine-adjusted BOE by tobacco use group (Table S4). Pairwise group differences in log-transformed creatinine-adjusted BOEs, adjusted for age, sex, race/ethnicity, and BMI, were tested in a weighted regression analysis. Adjusted geometric means were computed by exponentiating predicted population margins with covariate values fixed at the observed means. Contrast *t* tests assessed the null hypothesis that the adjusted geometric mean ratio (“GMR”) was equal to one.

**Figure 1:**
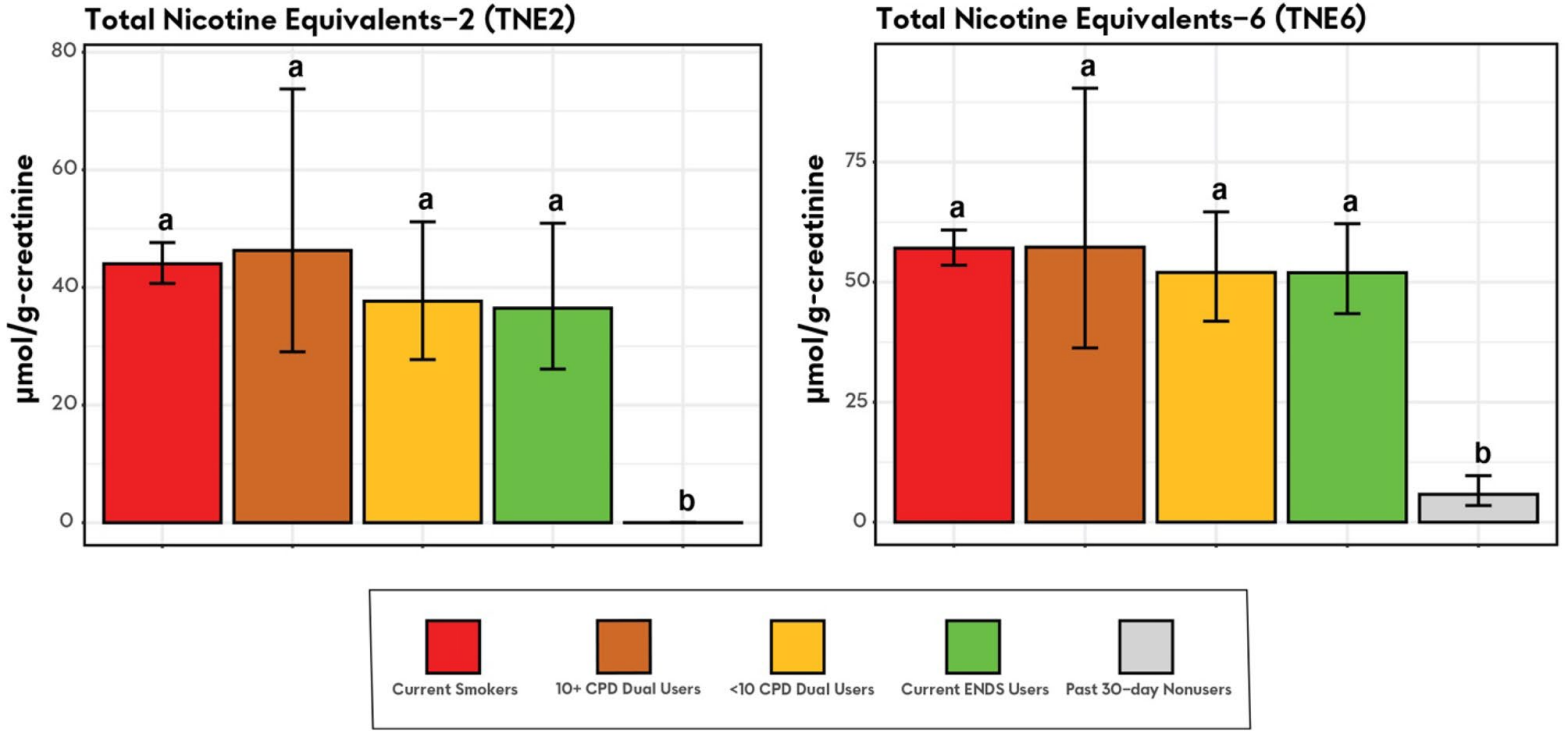
Nicotine equivalents among smokers, ENDS users, dual users stratified by cigarettes/day and tobacco nonusers (weighted adjusted geometric mean and 95% confidence interval). *Note*. Groups whose bars do not share a letter above the bar are significantly different (*p* < 0.05). Groups whose bars share a letter do not significantly differ from each other (*p* ≥ 0.05). Geometric mean ratios, and more exact p-values are shown in Table S6 in the Supplemental Materials. Adjusted geometric means and confidence interval endpoints were derived from a weighted regression analysis with covariates for age, sex, race/ethnicity, and BMI and were computed by exponentiating predicted population margins with covariate values fixed at the observed means. The analysis was weighted to represent the US adult civilian, noninstitutionalized population of never, current, and recent (within 1-year) former tobacco users. Current Smokers: TNE2, N = 1341; TNE6, N = 1327. 10 + CPD Dual Users: TNE2, N = 61; TNE6, N = 61. < 10 CPD Dual Users: TNE2, N = 54; TNE6, N = 53. Current ENDS Users: TNE2, N = 151; TNE6, N = 146. Past 30-day Nonusers: TNE2, N = 1842; TNE6, N = 152.

**Figure 2:**
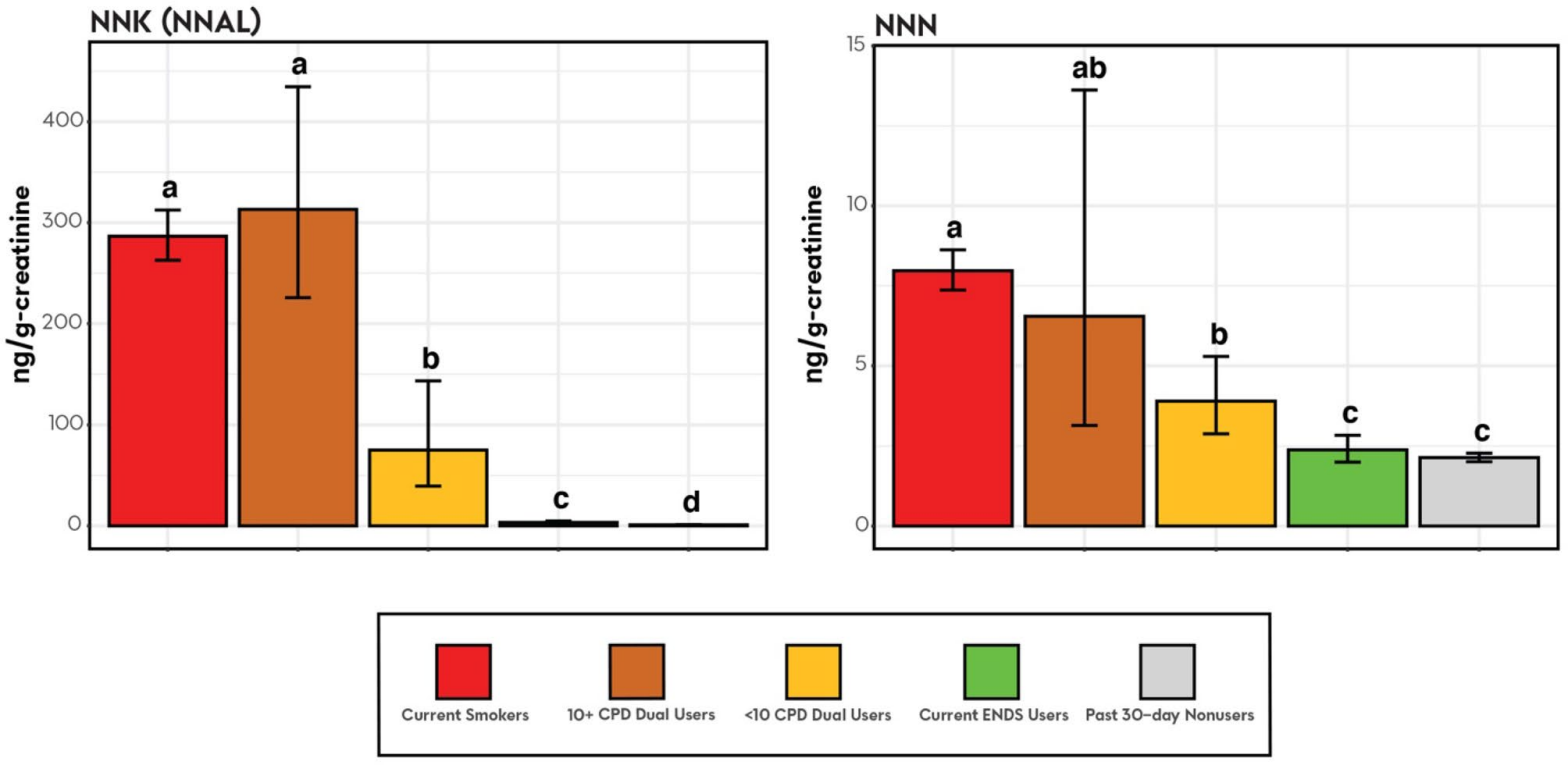
BOEs of tobacco-specific nitrosamines among smokers, ENDS users, dual users stratified by cigarettes/day and past 30-day nonusers (weighted adjusted geometric mean and 95% confidence interval). *Note*. Groups whose bars do not share a letter above the bar are significantly different (*p* < 0.05). Groups whose bars share a letter do not significantly differ from each other (*p* ≥ 0.05). Geometric mean ratios, and more exact p-values are shown in Table S6 in the Supplemental Materials. Adjusted geometric means and confidence interval endpoints were derived from a weighted regression analysis with covariates for age, sex, race/ethnicity, and BMI and were computed by exponentiating predicted population margins with covariate values fixed at the observed margins. The analysis was weighted to represent the US adult civilian, noninstitutionalized population of never, current, and recent (within 1-year) former tobacco users. Current Smokers: NNAL, N = 1338; NNN, N = 1309. 10 + CPD Dual Users: NNAL, N = 60; NNN, N = 58. < 10 CPD Dual Users: NNAL, N = 54; NNN, N = 53. Current ENDS Users: NNAL, N = 149; NNN, N = 148. Past 30-day Nonusers: NNAL, N = 1828; NNN, N = 1836.

**Figure 3:**
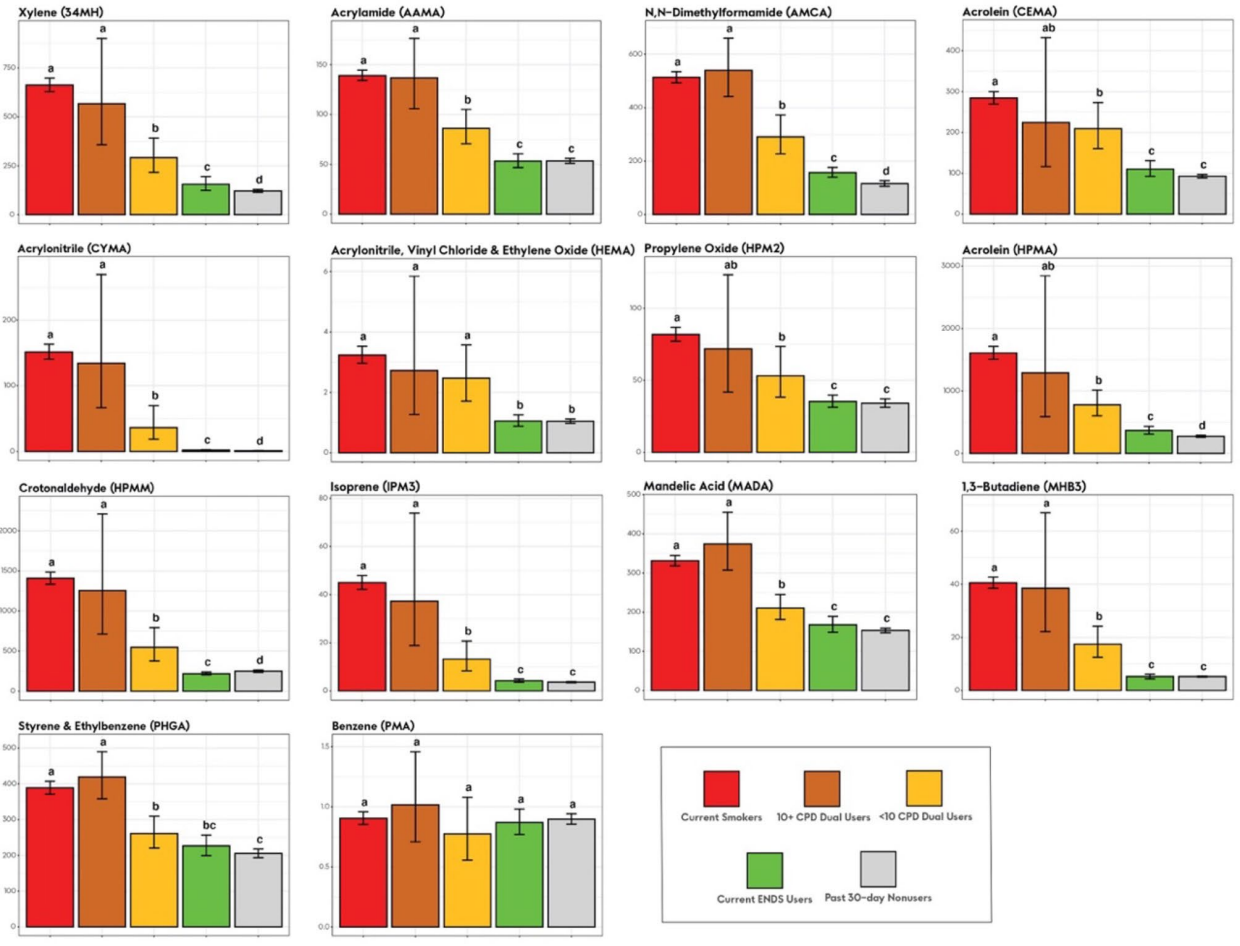
BOEs of VOCs among smokers, ENDS users, dual users stratified by CPD and past 30-day nonusers (weighted adjusted geometric mean and 95% confidence interval). *Note*. All values represent μg/g creatinine. Groups whose bars do not share a letter above the bar are significantly different (*p* < 0.05). Groups whose bars share a letter do not significantly differ from each other (*p* ≥ 0.05). Geometric mean ratios, and more exact p-values are shown in Table S6 in the Supplemental Materials. Adjusted geometric means and confidence interval endpoints were derived from a weighted regression analysis with covariates for age, sex, race/ethnicity, and BMI and were computed by exponentiating predicted population margins with covariate values fixed at the observed means. The analysis was weighted to represent the US adult civilian, noninstitutionalized population of never, current, and recent (within 1-year) former tobacco users. Current Smokers, N = 1341; 10 + CPD Dual Users, N = 61; < 10 CPD Dual Users, N = 54; ENDS Users, N = 151; Past 30-day Nonusers, N = 1846.

**Figure 4:**
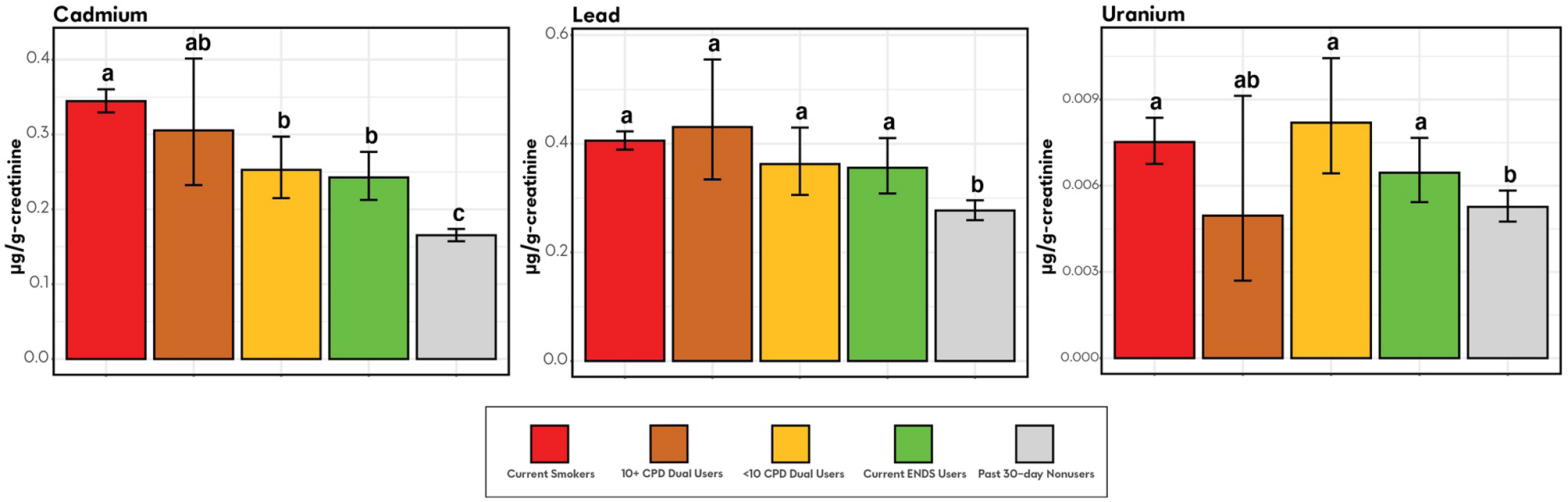
BOEs of metals among smokers, ENDS users, dual users stratified by cigarettes/day and past 30-day nonusers (weighted adjusted geometric mean and 95% confidence interval). *Note.* Groups whose bars do not share a letter above the bar are significantly different (*p* < 0.05). Groups whose bars share a letter do not significantly differ from each other (*p* ≥ 0.05). Geometric mean ratios, and more exact p-values are shown in Table S6 in the Supplemental Materials. Adjusted geometric means and confidence interval endpoints were derived from a weighted regression analysis with covariates for age, sex, race/ethnicity, and BMI and were computed by exponentiating predicted population margins with covariate values fixed at the observed means. The analysis was weighted to represent the US adult civilian, noninstitutionalized population of never, current, and recent (within 1-year) former tobacco users. Current Smokers, N = 1341; 10 + CPD Dual Users, N = 61; < 10 CPD Dual Users, N = 54; ENDS Users, N = 151; Past 30-day Nonusers, N = 1845.

Adjusted analyses of geometric mean creatinine-adjusted BOE levels with two Dual User strata are presented in Figs. 1, 2, 3, and 4. Unadjusted and adjusted analyses, and analyses with one Dual User stratum are presented in the Supplemental Materials.

All analyses were conducted using SAS© software (version 9.4, SAS Institute, Cary, NC, USA) and were weighted to represent the 2013–2014 U.S. adult civilian, noninstitutionalized population of never, current, and recent former (within 1-year) tobacco users with the Wave 5 single-wave weights for the Wave 1 Biomarker Core. Hypothesis tests used the 0.05 significance level without adjustment for multiple comparisons. Variance estimation used Fay’s Balanced Repeated Replication method with Fay’s factor set to 0.3 and 100 replicate weights to account for the complex survey design structure of the PATH Study. Estimates are reported for which the relative standard error (RSE) is greater than 30% or for which the proportion of subjects with measurements below the LOD exceeded 40%.

## Results

### Sociodemographic characteristics

On average, Smokers and Nonusers were older than Dual Users and ENDS Users (Table 2). Nearly three-quarters of Dual Users were female. Approximately 90% of ENDS Users had a history of established smoking (i.e., smoked more than 100 cigarettes). Dual Users smoked significantly fewer cigarettes/day (mean = 10.3 [*SE* = 0.93]) than Smokers (mean = 13.8 [*SE* = 0.39]). When stratified by median cigarettes/day, Dual Users formed two distinct groups: those who smoked less than 10 cigarettes per day (N = 54; “< 10 CPD Dual Users”) averaged 3.5 cigarettes/day (*SE* = 0.5) and those who smoked at least 10 cigarettes per day (N = 61; “10 + CPD Dual Users”) averaged 14.5 cigarettes/day (*SE* = 1.5). Past 30-day smoking and ENDS use behaviors differed in the two Dual User strata; the average 10 + CPD Dual User smoked nearly every day (29.5 of the past 30 days) and used ENDS on about half as many days (18.2 of the past 30 days), whereas < 10 CPD Dual Users used ENDS (24.0 of the past 30 days) more frequently than cigarettes (20.6 days of the past 30 days).

**Table 2:** Descriptive sociodemographic characteristics at Wave 5 (2018–2019).

Sample characteristic	Smokers (N = 1341)	Dual users (N = 115)	Dual users (≥ 10 CPD) (N = 61)^a^	Dual users (< 10 CPD) (N = 54)^a^	ENDS users (N = 151)	Tobacco nonusers (N = 1846)
Sociodemographic characteristics						
Age, mean (SE)	49.5 (0.66)	40.0 (2.60)	41.1 (4.2)	38.3 (1.7)	41.2 (1.50)	49.5 (0.43)
Sex, % (SE)						
Male	47.9 (1.87)	32.0 (7.16)	23.3 (7.81)	46.2 (9.90)	54.0 (6.02)	42.5 (1.45)
Female	52.1 (1.87)	68.0 (7.16)	76.7 (7.81)	53.8 (9.90)	46.0 (6.02)	57.5 (1.45)
Race/ethnicity, % (SE)						
Non-Hispanic White	67.7 (1.98)	90.1 (3.22)	90.0 (4.61)	90.3 (4.06)	81.4 (4.74)	60.2 (2.13)
Non-Hispanic Black	14.2 (1.49)	3.5 (1.78)	–	–	9.8 (4.19)	12.0 (1.22)
Hispanic	4.9 (0.81)	3.8 (2.05)	–	–	5.6 (2.11)	8.6 (1.28)
Non-Hispanic Other Race	13.2 (1.11)	2.6 (0.97)	–	–	3.1 (1.32)	19.2 (1.43)
BMI, mean (SE)	27.9 (0.15)	27.5 (0.58)	27.3 (0.6)	27.9 (1.0)	27.7 (0.49)	28.2 (0.23)
Cigarette smoking characteristics						
Former established smoker, % (SE)	–	–	–	–	88.5 (4.15)	12.2 (0.8)
CPD, mean (SE)	13.8 (0.39)	10.3 (0.93)^b^	14.5 (1.5)	3.5 (0.5)	–	–
Days smoked cigarettes in past 30 days, mean (SE)	28.0 (0.2)	26.1 (1.0)	29.5 (0.5)	20.6 (1.7)	–	–
Years since started smoking regularly, mean (SE)	31.3 (0.7)	23.3 (3.1)	24.2 (4.7)	21.5 (1.7)	–	–
ENDS use characteristics						
Days used ENDS in past 30 days, mean (SE)	–	20.4 (3.0)	18.2 (4.3)	24.0 (1.8)	28.3 (0.7)	–
ENDS device type most often used, % (SE)						
Open system	–	54.7 (10.21)	45.8 (14.17)	69.3 (7.81)	72.7 (4.81)	–
Closed system	–	45.3 (10.21)	54.2 (14.17)	30.7 (7.81)	27.3 (4.81)	–
ENDs flavor use in past 30 days, multi-choice, % (SE)						
Any tobacco flavor	–	17.2 (5.88)	14.7 (8.45)	21.3 (6.98)	15.3 (3.70)	–
Any menthol/mint, no tobacco	–	24.1 (6.56)	22.9 (9.77)	26.0 (7.96)	30.4 (4.83)	–
Exclusive other-flavor use, no tobacco or menthol or mint	–	58.7 (8.75)	62.4 (12.89)	52.7 (8.72)	54.2 (5.27)	–
Years since started using ENDS regularly, mean (SE)	–	5.8 (0.4)	5.5 (0.4)	6.1 (0.7)	6.2 (0.1)	–

Means, percentages, and standard errors were weighted to represent the US adult civilian, noninstitutionalized population of never, current, and recent (within 1-year) former tobacco users. Missing estimates (“–”) cannot be reported and/or were not submitted for disclosure clearance review. ^a^Tobacco use group is a subset of Dual users (N = 115). ^b^Dual users reported fewer average cigarettes per day (CPD) than CS (p < 0.01).

About three-quarters of ENDS Users used open systems and used ENDS on approximately 28 of the last 30 days, on average (Table 2). Over half of Dual Users and ENDS Users reported using non-Tobacco/Menthol/Mint flavors.

### Nicotine equivalents

Geometric mean levels of nicotine equivalents were significantly higher in in all nicotine-using groups than in Nonusers, and did not significantly differ between Smokers, ENDS Users and both Dual Users groups (Fig. 1). Levels of nicotine exposure in 10 + CPD Dual Users were more variable than levels in other nicotine-using groups.

### TSNAs

Geometric mean levels of the two available TSNA BOEs—NNAL and NNN—were significantly lower in ENDS Users than in Smokers (Fig. 2). Levels of NNAL were significantly higher in ENDS Users than in Nonusers, but levels of NNN did not significantly differ between ENDS Users and Nonusers. Levels of NNAL and NNN in < 10 CPD Dual Users were significantly lower than levels in Smokers and significantly greater than levels in ENDS Users. Dual Users who smoked 10 or more cigarettes/day did not significantly differ from Smokers. The magnitude of differences in geometric mean NNAL levels are substantial: NNAL levels in all smoking groups were at least 22 times greater than levels in ENDS Users, with levels in Smokers nearly 86 times greater than levels in ENDS Users (Tables S5 and S6). In contrast, NNAL levels in ENDS Users were 3.5 times greater than levels in Nonusers.

### BOEs of VOCs

Geometric mean levels of PMA, a marker for benzene exposure, did not significantly differ in any of the study groups (Fig. 3); therefore PMA was not considered further, leaving 13 VOC BOEs for subsequent comparisons.

Levels of all 13 VOC BOEs were significantly lower in ENDS Users than in Smokers, and for 8 of 13 VOC BOEs, levels in ENDS Users did not significantly differ from Nonusers (Fig. 3). VOC BOE levels in < 10 CPD Dual Users were intermediate between Smokers’ and ENDS Users’; for 12 of 13 VOC BOEs, levels in < 10 CPD Dual Users were significantly lower than in Smokers, but significantly greater than in ENDS users (Fig. 3). VOC BOE levels in 10 + CPD Dual Users did not significantly differ from levels in Smokers (Fig. 3).

### Metals

Geometric mean levels for all three metals were significantly higher among Smokers and ENDS Users than Nonusers (Fig. 4). Levels of one metal—cadmium—were significantly lower in ENDS Users and Dual Users who smoked < 10 cigarettes/day than in Smokers (Fig. 4). In contrast, levels in Dual Users who smoked ≥ 10 cigarettes/day did not significantly differ from levels in Smokers.

## Discussion

In this nationally-representative observational study of US adults, exclusive users of ENDS showed equivalent levels of nicotine, but substantially lower levels of TSNAs, VOCs, and one metal compared to cigarette smokers. Exclusive ENDS users did not significantly differ from past 30-day tobacco nonusers in inferred exposure to many TSNAs and VOCs, and in many cases the observed BOE values were very similar in magnitude. Overall, the BOE data demonstrate that exclusive ENDS users were able to obtain similar levels of nicotine as cigarette smokers but with significantly lower levels of exposure to numerous toxicants, including carcinogens.

The results of this analysis concord with previous observational studies using data from the PATH Study^24–26^ and other studies^39^ that were conducted when earlier-generation ENDS products were predominant, and extend these findings to the more recent tobacco product marketplace, specifically recent-generation ENDS products. The levels of BOEs observed among ENDS users relative to cigarette smokers are also consistent with controlled confinement studies, randomized trials and longitudinal observational studies in which smokers who switch to ENDS experience substantial reductions in BOEs^10,40,41^, and suggest that smokers who switch to exclusive ENDS use can reduce exposure to many HPHCs for which biomarker data are available to a similar extent as abstinence from tobacco products or smoking cessation. Although the dose–response relationship between HPHC exposures and subsequent disease are complex (e.g., subject to threshold effects), lower exposures are likely to be associated with lower disease risk, implying that smokers who switch to ENDS would likely experience decreased disease risk^5,6^.

Dual Users were extremely heterogeneous in exposure, and partitioning by cigarette consumption showed that the key driver of toxic exposure was the quantity of cigarette consumption. Some analyses have reported that dual users of both ENDS and cigarettes have exposures as high or even higher than those seen in exclusive smokers^42–44^. In the present analysis, levels of both TSNA and 12 of 13 VOC BOEs were significantly lower in dual users reporting smoking < 10 cigarettes/day than in exclusive cigarette smokers, though levels of all BOEs assessed were significantly greater in < 10 CPD Dual Users than in Nonusers.

Conversely, Dual Users smoking 10 or more cigarettes/day—whose average cigarette consumption was similar to that seen in Smokers (mean CPD = 14.5 vs. 13.8), showed levels of BOEs that were not significantly lower than Smokers’ levels but higher than ENDS Users and Nonusers. Thus, the results indicate that the BOE levels observed in Dual Users are largely attributable to Dual Users’ cigarette smoking. This dose–exposure association is consistent with previous analyses of the PATH Study showing that cigarette smoking is the primary driver of HPHC exposure among dual users^24,25^, with HPHC exposure increasing with increasing cigarette consumption^27,45^.

The findings regarding dual use are also consistent with controlled clinical confinement studies and randomized trials demonstrating that dual users who reduce their daily cigarette consumption by at least 50% significantly reduce their HPHC exposure compared to smokers who continue smoking as usual^40,46–53^. These data regarding HPHC exposure among dual users are especially pertinent given that dual use is a common initial use pattern among smokers who adopt ENDS, although it often serves as a transitionary state on the pathway to complete switching away from smoking^54^, ultimately reducing exposures further.

These findings regarding actual human exposure are consistent with chemical analyses of aerosols produced by ENDS under conditions that mimic real-world use, which show that these TSNAs and VOCs are absent or present at levels much lower than in cigarette smoke^9^. It is logical that the chemical composition of the ENDS aerosol would be predictive of the users’ ultimate exposure, as users only inhale chemicals present in the aerosol matrix. This suggests that chemical analyses of the aerosols can be used to infer human exposures, which is particularly important since some constituents that can be measured in the aerosols do not have validated biomarkers.

Chemical analyses of ENDS aerosols have found levels of metals above established toxicological standards^18,19,55,56^. However, many of these experiments have been criticized for using procedures that do not replicate real world patterns of ENDS use and other methodological limitations (e.g., over-powering or overheating the coil)^57^. The results of the current study demonstrate that, on average, ENDS Users are exposed to lower levels of cadmium than Smokers. Future naturalistic research is needed to assess levels of other metals beyond the three included in the PATH Study. Exposure to many VOCs can occur from sources other than tobacco use, and thus nonusers represent the benchmark for baseline levels of environmental exposures to such VOCs^8^. For example, acrylamide exposure can occur from many foods as well as from tobacco use^58,59^, and the levels observed in ENDS users were almost exactly equivalent to those in nonusers of tobacco. PMA, a marker for benzene exposure, was not elevated in Smokers (vs. Nonusers), and showed similar levels across all groups, suggesting that benzene exposure was occurring primarily from environmental sources, rather than tobacco product use.

All the nicotine-using groups (i.e., Smokers, ENDS Users and both Dual User strata) demonstrated similar levels of nicotine exposure, all significantly higher than those in tobacco product nonusers. This is consistent with the concept that ENDS are intended as alternative nicotine sources to allow smokers who are not quitting to maintain nicotine intake, while reducing exposure to HPHCs associated with cigarette smoking^60,61^. Used as a harm-reduction strategy, ENDS are intended to draw smokers away from combustible cigarettes, which are the most harmful and addictive nicotine-delivery product. Consistent with these conceptual models of switching and nicotine delivery, data from observational studies^62^ and randomized trials^63,64^ support the concept that adequate levels of nicotine are likely necessary to facilitate switching away from smoking.

Strengths of the study include the representative sample of US adults, large overall sample size and use of a dataset that includes more recent-generation ENDS devices. A limitation of the current analysis was that its measures of exposure were limited to select TSNAs, VOCs, three heavy metals, and nicotine. Other HPHCs such as polycyclic aromatic hydrocarbons (PAHs) were not available from PATH Study Wave 5 at the time of analysis (Version 18). The BOEs currently available from the PATH Study also did not include markers for exposures that might be higher in ENDS users. Finally, the analysis was a cross-sectional, between-groups comparison, so it does not directly show a within-participants reduction in HPHCs among smokers who switch to ENDS.

In summary, this nationally-representative analysis of a large sample of US adults found that exclusive users of ENDS have significantly and substantially lower exposures to a range of TSNAs, VOCs and one metal compared to smokers, with exposures among ENDS users often comparable to those seen in individuals not using any tobacco products at all. Such reductions in exposure have been taken as evidence of reductions in risk of smoking-induced disease^9–11^. In concert with the results of randomized clinical trials and longitudinal studies, the current results indicate that smokers who switch to ENDS likely experience reductions in health risk. The data also indicate that exposure to TSNAs, VOCs and metals among dual users was primarily a function of their cigarette smoking, as dual users who were smoking fewer than 10 cigarettes a day also consistently showed lower exposures compared to smokers. However, the data make clear that complete switching, with no smoking at all, results in reduced exposures compared to dual use with low levels of smoking. Further research on other toxicants, and also on biomarkers of potential harm, such as markers of inflammation, can inform reductions in health risks likely to accompany switching from smoking cigarettes to ENDS.

## Supplementary Information

Supplementary Information.

## Data Availability

The PATH Study datasets analyzed in this study are publicly available via application to the Inter-university Consortium for Political and Social Research (ICPSR) Virtual Data Enclave. Details are provided for the BRUF and RUF at 10.3886/ICPSR36840.v18 and 10.3886/ICPSR36231.v31, respectively.

## Code availability

SAS code used to generate results for the current study are available in the Supplemental Code file.
