# Supplementary material for: Comparison of biomarkers of exposure among US adult smokers, users of electronic nicotine delivery systems, dual users and nonusers, 2018–2019
Source: Sci Rep. 2023 May 5;13:7297. doi: 10.1038/s41598-023-34427-x (PMC10163269; doi:10.1038/s41598-023-34427-x)
Supplement: Supplementary file 1 — Supplementary Information. [file 41598_2023_34427_MOESM1_ESM.pdf]

Table S1. Analysis Subpopulation

PATH Respondent Subset	Included (N)
Completed Wave 5 Adult Interview Questionnaire	34,309
Provided urine sample at Wave 5	12,102
Valid Wave 5 Single-Wave Weight for the Wave 1 Biomarker Core	7,868
Final Analytic Sample	3,453

Source: PATH Study Wave 5 Restricted-Use Files and Biomarker Restricted-Use Files
Note: N=unweighted sample size. "Completed Wave 5 Adult Interview Questionnaire" is the case count in the publicly-available documentation file 36840-5001-Codebook.pdf. "Provided a urine sample at Wave 5" is a case count for the variable R05R_A_Biocollect_Urine in the publicly-available documentation file 36840-5001-Codebook.pdf. "Valid Wave 5 Single-Wave Weight for the Wave 1 Biomarker Core" is the count of valid cases in the publicly-available documentation file 36840-5022-Codebook.pdf.

Table S2. Unweighted CPD Summary Statistics for Smokers and Dual Users

Tobacco Use Group	Unweighted CPD Summary Statistic				
	N	25th Percentile	50th Percentile	75th Percentile	Mean (*SE*)
Smokers	1341	6.67	10.27	20	13.6 (0.26)
Dual Users	115	4	10	15	9.8 (0.73)
Dual Users ≥10 cigarettes/day	61	—	15	—	15.4 (0.83)
Dual Users <10 cigarettes/day	54	—	4	—	3.6 (0.40)

Source: PATH Study Wave 5 Restricted-Use Files and Biomarker Restricted-Use Files
Note: N=unweighted sample size; SE=Standard Error

Table S3. Proportion of Measurements Below the Lower Limit of Detection (LOD) by Tobacco Use Group, Selected BOEs

		Unweighted Proportion < LOD (Weighted Proportion < LOD)			
**Biomarker, $\left(\frac{ng}{mL}\right)$**	**Abbreviation**	**CS (N=1341)**	**DU (N=115)**	**CE (N=151)**	**NU (N=1846)**
N-Acetyl-S-(2-hydroxyethyl)-L-cysteine	HEMA	15.1 (16.5)	19.1 (11.8)	52.3 (54.9)	49.2 (54.1)
N-Acetyl-S-(phenyl)-L-cysteine	PMA	43.5 (46.1)	49.6 (45.4)	43.0 (37.2)	42.4 (42.4)
4-(methylnitrosamino)-1-(3-pyridyl)-1-butanol	NNAL	0.0 (0.0)	0.0 (0.0)	15.9 (15.1)	36.6 (45.1)
N'-Nitrosonornicotine	NNN	18.9 (18.1)	30.4 (36.1)	83.4 (83.7)	96.8 (98.7)
Uranium, $\left(\frac{\mu g}{L}\right)$	UUR	20.0 (20.1)	20.9 (35.4)	19.2 (18.6)	23.1 (24.1) [a]

Source: PATH Study Wave 5 Restricted-Use Files and Biomarker Restricted-Use Files
Note: Weighted estimates were weighted to represent the US adult civilian, noninstitutionalized population of never, current, and recent (within 1-year) former tobacco users. [a] Sample size varied by BOE. NU: N=1845. CS=Current Exclusive Cigarette Smoker; DU=Current Dual User; CE=Current Exclusive ENDS User; NU=Past 30-day nonuser; ENDS=Electronic Nicotine Delivery System; PATH=Population Assessment of Tobacco and Health; LOD=Limit of Detection; N=unweighted sample size.

Table S4. Weighted Geometric Mean Creatinine-adjusted Biomarker Concentration

Creatinine-adjusted Biomarker, $\left(\frac{\mu g}{g-cr}\right)$	Abbreviation	Weighted Geometric Mean (95% Confidence Interval) CS (N=1341)	DU (N=115)	DU+10 (N=61) [a]	DU-10 (N=54) [a]	CE (N=151)	NU (N=1846)
3-Methylhippuric acid + 4-Methylhippuric acid	34MH	692.3 (657.2, 729.2)	478.6 (356.1, 643.3)	623.9 (371.9, 1047.0)	309.8 (228.8, 419.5)	159.9 (130.4, 196.1)	125.2 (117.9, 133.0)
N-Acetyl-S-(2-carbamoylethyl)-L-cysteine	AAMA	139.0 (133.9, 144.2)	119.5 (102.8, 138.9)	144.1 (112.2, 185.1)	87.9 (72.5, 106.6)	53.4 (46.5, 61.2)	53.5 (50.9, 56.3)
N-Acetyl-S-(N-methylcarbamoyl)-L-cysteine	AMCA	540.6 (515.4, 567.1)	472.7 (387.6, 576.5)	617.1 (477.9, 796.7)	305.2 (233.0, 399.9)	157.0 (136.6, 180.5)	122.0 (111.9, 132.9)
N-Acetyl-S-(2-carboxyethyl)-L-cysteine	CEMA	294.6 (279.6, 310.4)	211.6 (134.8, 332.0)	219.6 (102.4, 470.9)	199.0 (151.9, 260.6)	107.5 (89.1, 129.6)	96.6 (91.7, 101.7)
N-Acetyl-S-(2-cyanoethyl)-L-cysteine	CYMA	151.4 (141.3, 162.3)	83.5 (54.4, 128.0)	138.5 (66.8, 287.2)	36.4 (19.3, 68.5)	2.09 (1.62, 2.71)	1.23 (1.17, 1.29)
N-Acetyl-S-(2-hydroxyethyl)-L-cysteine	HEMA	3.16 (2.904, 3.449)	2.73 (1.729, 4.314)	2.9 (1.3, 6.5)	2.4 (1.7, 3.4)	1.02 (0.842, 1.231)	1.04 (0.978, 1.116)
N-Acetyl-S-(2-hydroxypropyl)-L-cysteine	HPM2	80.5 (76.0, 85.2)	69.1 (51.2, 93.3)	79.6 (47.1, 134.4)	54.9 (41.0, 73.5)	35.1 (31.4, 39.2)	33.9 (31.0, 37.2)
N-Acetyl-S-(3-hydroxypropyl)-L-cysteine	HPMA	1628.0 (1534.0, 1728.0)	1074.3 (665.6, 1734.0)	1312.0 (545.5, 3155.0)	773.9 (602.3, 994.4)	366.2 (309.2, 433.8)	280.3 (263.2, 298.4)
N-Acetyl-S-(3-hydroxypropyl-1-methyl)-L-cysteine	HPMM	1489.8 (1407.0, 1578.0)	955.2 (660.5, 1382.0)	1342.5 (698.0, 2582.0)	546.4 (383.6, 778.3)	220.0 (199.0, 243.2)	264.5 (248.7, 281.3)
N-Acetyl-S-(4-hydroxy-2-methyl-2-buten-1-yl)-L-cysteine	IPM3	47.1 (44.0, 50.4)	27.4 (17.6, 42.6)	41.6 (19.3, 89.6)	13.8 (8.9, 21.5)	4.33 (3.73, 5.03)	3.75 (3.48, 4.03)
Mandelic acid	MADA	338.4 (324.5, 352.8)	312.5 (261.5, 373.5)	396.2 (320.0, 490.6)	211.7 (183.1, 244.9)	166.7 (149.1, 186.4)	157.1 (150.9, 163.7)
N-Acetyl-S-(4-hydroxy-2-buten-1-yl)-L-cysteine	MHB3	42.0 (39.8, 44.3)	29.6 (20.9, 41.8)	40.8 (22.0, 75.5)	17.5 (12.6, 24.2)	5.17 (4.39, 6.09)	5.35 (5.16, 5.56)
Phenylglyoxylic acid	PHGA	399.8 (380.9, 419.6)	363.8 (320.6, 412.8)	440.0 (372.5, 519.7)	266.3 (223.7, 317.0)	228.5 (202.1, 258.2)	210.3 (197.7, 223.6)
N-Acetyl-S-(phenyl)-L-cysteine	PMA	0.930 (0.875, 0.989)	0.968 (0.729, 1.286)	1.094 (0.767, 1.562)	0.792 (0.564, 1.112)	0.876 (0.764, 1.004)	0.927 (0.882, 0.975)
4-(methylnitrosamino)-1-(3-pyridyl)-1-butanol, $\left(\frac{ng}{g-cr}\right)$	NNAL	297.1 (272.9, 323.4)	193.0 (134.5, 277.0)	339.1 (237.9, 483.4)	77.1 (40.6, 146.4)	3.42 (2.37, 4.94)	0.97 (0.89, 1.06)

N'-Nitrosonornicotine, $\left(\frac{ng}{g-cr}\right)$	NNN	8.30<br>(7.68, 8.96)	5.85<br>(3.75, 9.11)	7.33<br>(3.21, 16.74)	4.05<br>(3.04, 5.40)	2.42<br>(1.98, 2.95)	2.23<br>(2.10, 2.38)
Cadmium	UCD	0.391<br>(0.365, 0.419)	0.273<br>(0.211, 0.353)	0.316<br>(0.207, 0.481)	0.215<br>(0.168, 0.275)	0.217<br>(0.176, 0.268)	0.193<br>(0.183, 0.204)
Lead	UPB	0.440<br>(0.418, 0.464)	0.373<br>(0.303, 0.460)	0.414<br>(0.290, 0.590)	0.316<br>(0.264, 0.377)	0.326<br>(0.270, 0.393)	0.309<br>(0.288, 0.330)
Uranium	UUR	0.008<br>(0.007, 0.008)	0.006<br>(0.004, 0.010)	0.005<br>(0.003, 0.010)	0.008<br>(0.006, 0.010)	0.006<br>(0.005, 0.008)	0.006<br>(0.005, 0.006)
Total Nicotine Equivalents-2, $\left(\frac{\mu mol}{g-cr}\right)$	TNE2	43.4<br>(40.5, 46.6)	45.5<br>(34.5, 60.1)	50.1<br>(32.3, 77.8)	38.9<br>(28.4, 53.1)	36.8<br>(26.5, 51.0)	0.004<br>(0.003, 0.005)
Total Nicotine Equivalents-6, $\left(\frac{\mu mol}{g-cr}\right)$	TNE6	62.7<br>(59.1, 66.5)	61.3<br>(44.6, 84.3)	64.5<br>(38.1, 109.2)	56.3<br>(44.9, 70.5)	55.9<br>(46.4, 67.4)	5.44<br>(3.22, 9.20)

Source: PATH Study Wave 5 Restricted-Use Files and Biomarker Restricted-Use Files
Note: Sample size varied by BOE. CS: NNAL, N=1338; NNN, N=1309; TNE6, N=1327. DU: NNAL, TNE6, N=114; NNN, N=111. DU+10: NNAL, N=60; NNN, N=58. DU-10: NNN, TNE6, N=53. CE: NNAL, N=149; NNN, N=148; TNE6, N=146. NU: NNAL, N=1828; NNN, N=1836; UCD, UPB, UUR, N=1845; TNE2, N=1842; TNE6, N=152.
[a] Tobacco use group is a subset of DU (N=115) presented in this table.
Estimates and confidence interval endpoints was weighted to represent the US adult civilian, noninstitutionalized population of never, current, and recent (within 1-year) former tobacco users. CS=Current Exclusive Cigarette Smoker; DU=Current Dual User; DU+10=DU reporting 10+ CPD; DU-10=DU reporting <10 CPD; CE=Current Exclusive ENDS User; NU=Past 30-day nonuser; ENDS=Electronic Nicotine Delivery System; PATH=Population Assessment of Tobacco and Health; N=unweighted sample size; SE=Standard Error; g-cr=grams, urinary creatinine.

Table S5. Weighted Unadjusted and Adjusted Geometric Mean Ratio by Creatinine-adjusted Biomarker, One Dual User Stratum

Creatinine-adjusted Biomarker	Unadjusted GMR	95% CI	p-Value	Adjusted GMR	95% CI	p-Value
Smokers vs. Dual Users						
34MH	1.45	(1.08, 1.93)	0.0131	1.50	(1.14, 1.98)	0.0044
AAMA	1.16	(1.00, 1.36)	0.0532	1.21	(1.04, 1.42)	0.0154
AMCA	1.14	(0.94, 1.39)	0.1830	1.20	(1.03, 1.41)	0.0229
CEMA	1.39	(0.89, 2.17)	0.1437	1.30	(0.86, 1.96)	0.2078
CYMA	1.81	(1.19, 2.76)	0.0058	1.85	(1.20, 2.84)	0.0055
HEMA	1.16	(0.74, 1.81)	0.5121	1.23	(0.77, 1.98)	0.3856
HPM2	1.16	(0.86, 1.58)	0.3255	1.28	(0.92, 1.78)	0.1448
HPMA	1.52	(0.95, 2.41)	0.0795	1.51	(0.95, 2.38)	0.0793
HPMM	1.56	(1.09, 2.24)	0.0160	1.54	(1.10, 2.16)	0.0132
IPM3	1.72	(1.12, 2.63)	0.0131	1.79	(1.18, 2.71)	0.0066
MADA	1.08	(0.90, 1.31)	0.4058	1.10	(0.93, 1.31)	0.2680
MHB3	1.42	(1.01, 1.99)	0.0433	1.42	(1.03, 1.97)	0.0349
PHGA	1.10	(0.96, 1.26)	0.1673	1.11	(0.98, 1.26)	0.1125
PMA	0.96	(0.71, 1.31)	0.7959	0.99	(0.73, 1.33)	0.9249
NNAL	1.54	(1.09, 2.18)	0.0160	1.58	(1.12, 2.21)	0.0090
NNN	1.42	(0.93, 2.17)	0.1039	1.48	(0.97, 2.26)	0.0671
UCD	1.43	(1.11, 1.85)	0.0066	1.21	(1.02, 1.44)	0.0276
UPB	1.18	(0.96, 1.45)	0.1193	1.01	(0.86, 1.18)	0.9492
UUR	1.22	(0.81, 1.85)	0.3338	1.25	(0.81, 1.93)	0.3000
TNE2	0.95	(0.72, 1.26)	0.7345	1.03	(0.75, 1.41)	0.8616
TNE6	1.02	(0.75, 1.40)	0.8865	1.03	(0.78, 1.37)	0.8225
Smokers vs. ENDS Users						
34MH	4.33	(3.46, 5.41)	<.0001	4.26	(3.35, 5.41)	<.0001
AAMA	2.60	(2.26, 3.01)	<.0001	2.61	(2.28, 2.99)	<.0001
AMCA	3.44	(2.97, 3.99)	<.0001	3.27	(2.88, 3.72)	<.0001
CEMA	2.74	(2.27, 3.32)	<.0001	2.58	(2.16, 3.09)	<.0001
CYMA	72.34	(54.94, 95.26)	<.0001	72.49	(55.06, 95.45)	<.0001
HEMA	3.11	(2.54, 3.81)	<.0001	3.08	(2.55, 3.72)	<.0001
HPM2	2.29	(2.03, 2.60)	<.0001	2.32	(2.04, 2.64)	<.0001
HPMA	4.45	(3.70, 5.35)	<.0001	4.38	(3.65, 5.26)	<.0001
HPMM	6.77	(6.04, 7.59)	<.0001	6.40	(5.76, 7.10)	<.0001
IPM3	10.87	(9.05, 13.07)	<.0001	10.66	(8.75, 12.98)	<.0001
MADA	2.03	(1.80, 2.29)	<.0001	1.98	(1.74, 2.25)	<.0001
MHB3	8.12	(6.77, 9.75)	<.0001	7.82	(6.46, 9.47)	<.0001

PHGA	1.75	(1.52, 2.01)	<.0001	1.72	(1.49, 1.99)	<.0001
PMA	1.06	(0.92, 1.22)	0.3911	1.04	(0.92, 1.18)	0.5414
NNAL	86.82	(59.81, 126.01)	<.0001	85.60	(59.04, 124.13)	<.0001
NNN	3.43	(2.81, 4.18)	<.0001	3.35	(2.79, 4.01)	<.0001
UCD	1.80	(1.44, 2.26)	<.0001	1.42	(1.23, 1.64)	<.0001
UPB	1.35	(1.12, 1.63)	0.0017	1.14	(0.99, 1.31)	0.0681
UUR	1.25	(1.02, 1.53)	0.0353	1.17	(0.97, 1.41)	0.1058
TNE2	1.18	(0.85, 1.63)	0.3137	1.21	(0.86, 1.69)	0.2728
TNE6	1.12	(0.93, 1.36)	0.2315	1.10	(0.91, 1.32)	0.3176
Smokers vs. Nonusers						
34MH	5.53	(5.11, 5.98)	<.0001	5.44	(5.06, 5.86)	<.0001
AAMA	2.60	(2.43, 2.78)	<.0001	2.61	(2.44, 2.78)	<.0001
AMCA	4.43	(4.02, 4.89)	<.0001	4.45	(4.02, 4.92)	<.0001
CEMA	3.05	(2.84, 3.28)	<.0001	3.06	(2.86, 3.29)	<.0001
CYMA	123.42	(113.43, 134.29)	<.0001	123.40	(113.13, 134.61)	<.0001
HEMA	3.03	(2.69, 3.42)	<.0001	3.10	(2.75, 3.49)	<.0001
HPM2	2.37	(2.13, 2.64)	<.0001	2.40	(2.15, 2.68)	<.0001
HPMA	5.81	(5.33, 6.33)	<.0001	5.85	(5.35, 6.39)	<.0001
HPMM	5.63	(5.20, 6.10)	<.0001	5.62	(5.19, 6.08)	<.0001
IPM3	12.58	(11.36, 13.93)	<.0001	12.38	(11.21, 13.67)	<.0001
MADA	2.15	(2.03, 2.28)	<.0001	2.16	(2.04, 2.29)	<.0001
MHB3	7.84	(7.33, 8.39)	<.0001	7.84	(7.36, 8.36)	<.0001
PHGA	1.90	(1.76, 2.05)	<.0001	1.89	(1.75, 2.05)	<.0001
PMA	1.00	(0.92, 1.09)	0.9361	1.01	(0.93, 1.09)	0.8431
NNAL	306.44	(274.65, 341.89)	<.0001	301.55	(270.05, 336.70)	<.0001
NNN	3.72	(3.33, 4.14)	<.0001	3.72	(3.35, 4.14)	<.0001
UCD	2.02	(1.87, 2.19)	<.0001	2.08	(1.95, 2.23)	<.0001
UPB	1.43	(1.31, 1.55)	<.0001	1.46	(1.35, 1.59)	<.0001
UUR	1.39	(1.24, 1.55)	<.0001	1.43	(1.27, 1.60)	<.0001
TNE2	10807	(9206, 12687)	<.0001	10,681	(9066, 12584)	<.0001
TNE6	11.52	(7.02, 18.90)	<.0001	9.83	(5.81, 16.66)	<.0001
Dual Users vs. ENDS Users						
34MH	2.99	(2.05, 4.37)	<.0001	2.83	(1.96, 4.11)	<.0001
AAMA	2.24	(1.83, 2.74)	<.0001	2.15	(1.76, 2.63)	<.0001
AMCA	3.01	(2.36, 3.84)	<.0001	2.72	(2.22, 3.34)	<.0001
CEMA	1.97	(1.22, 3.17)	0.0057	1.98	(1.27, 3.09)	0.0027
CYMA	39.88	(23.71, 67.07)	<.0001	39.19	(23.28, 65.97)	<.0001
HEMA	2.68	(1.65, 4.37)	0.0001	2.50	(1.50, 4.15)	0.0006

HPM2	1.97	(1.42, 2.73)	<.0001	1.82	(1.27, 2.59)	0.0012
HPMA	2.93	(1.80, 4.79)	<.0001	2.91	(1.80, 4.70)	<.0001
HPMM	4.34	(2.97, 6.35)	<.0001	4.16	(2.91, 5.95)	<.0001
IPM3	6.33	(3.96, 10.11)	<.0001	5.96	(3.74, 9.48)	<.0001
MADA	1.87	(1.49, 2.35)	<.0001	1.79	(1.45, 2.22)	<.0001
MHB3	5.72	(3.88, 8.45)	<.0001	5.50	(3.77, 8.03)	<.0001
PHGA	1.59	(1.31, 1.94)	<.0001	1.55	(1.28, 1.87)	<.0001
PMA	1.11	(0.81, 1.52)	0.5304	1.05	(0.77, 1.44)	0.7347
NNAL	56.40	(33.79, 94.16)	<.0001	54.32	(33.07, 89.22)	<.0001
NNN	2.42	(1.52, 3.83)	0.0003	2.26	(1.44, 3.52)	0.0005
UCD	1.26	(0.92, 1.72)	0.1477	1.17	(0.94, 1.46)	0.1584
UPB	1.15	(0.87, 1.52)	0.3359	1.13	(0.91, 1.41)	0.2598
UUR	1.02	(0.64, 1.62)	0.9342	0.93	(0.58, 1.49)	0.7598
TNE2	1.24	(0.80, 1.90)	0.3284	1.17	(0.75, 1.84)	0.4819
TNE6	1.10	(0.76, 1.58)	0.6178	1.06	(0.77, 1.47)	0.7116
Dual Users vs. Nonusers						
34MH	3.82	(2.84, 5.14)	<.0001	3.62	(2.73, 4.80)	<.0001
AAMA	2.23	(1.91, 2.62)	<.0001	2.15	(1.83, 2.52)	<.0001
AMCA	3.87	(3.14, 4.79)	<.0001	3.70	(3.06, 4.47)	<.0001
CEMA	2.19	(1.39, 3.46)	0.0009	2.36	(1.55, 3.59)	0.0001
CYMA	68.04	(44.75, 103.46)	<.0001	66.71	(43.34, 102.68)	<.0001
HEMA	2.61	(1.67, 4.10)	<.0001	2.51	(1.55, 4.08)	0.0003
HPM2	2.04	(1.49, 2.79)	<.0001	1.88	(1.34, 2.64)	0.0004
HPMA	3.83	(2.40, 6.12)	<.0001	3.88	(2.44, 6.18)	<.0001
HPMM	3.61	(2.51, 5.21)	<.0001	3.65	(2.59, 5.16)	<.0001
IPM3	7.32	(4.72, 11.35)	<.0001	6.92	(4.50, 10.65)	<.0001
MADA	1.99	(1.66, 2.39)	<.0001	1.96	(1.66, 2.32)	<.0001
MHB3	5.53	(3.91, 7.80)	<.0001	5.52	(3.96, 7.69)	<.0001
PHGA	1.73	(1.50, 2.00)	<.0001	1.71	(1.48, 1.97)	<.0001
PMA	1.04	(0.77, 1.41)	0.7757	1.02	(0.77, 1.36)	0.8799
NNAL	199.09	(135.92, 291.60)	<.0001	191.33	(131.89, 277.55)	<.0001
NNN	2.62	(1.71, 4.02)	<.0001	2.51	(1.64, 3.85)	<.0001
UCD	1.41	(1.09, 1.84)	0.0102	1.72	(1.44, 2.05)	<.0001
UPB	1.21	(0.98, 1.49)	0.0698	1.46	(1.23, 1.73)	<.0001
UUR	1.13	(0.75, 1.71)	0.5449	1.14	(0.74, 1.75)	0.5480
TNE2	11329	(8289, 15485)	<.0001	10389	(7231, 14925)	<.0001
TNE6	11.26	(6.35, 19.96)	<.0001	9.52	(5.26, 17.25)	<.0001
ENDS Users vs. Nonusers						

34MH	1.28	(1.03, 1.58)	0.0271	1.28	(1.01, 1.61)	0.0376
AAMA	1.00	(0.86, 1.16)	0.9737	1.00	(0.86, 1.16)	0.9717
AMCA	1.29	(1.10, 1.51)	0.0018	1.36	(1.17, 1.58)	<.0001
CEMA	1.11	(0.92, 1.34)	0.2636	1.19	(0.99, 1.42)	0.0585
CYMA	1.71	(1.32, 2.20)	<.0001	1.70	(1.33, 2.19)	<.0001
HEMA	0.97	(0.80, 1.18)	0.7912	1.01	(0.84, 1.21)	0.9456
HPM2	1.03	(0.89, 1.19)	0.6568	1.03	(0.89, 1.21)	0.6648
HPMA	1.31	(1.10, 1.56)	0.0033	1.34	(1.12, 1.59)	0.0016
HPMM	0.83	(0.74, 0.94)	0.0028	0.88	(0.79, 0.98)	0.0225
IPM3	1.16	(0.99, 1.36)	0.0745	1.16	(0.96, 1.40)	0.1125
MADA	1.06	(0.95, 1.18)	0.2861	1.09	(0.97, 1.23)	0.1431
MHB3	0.97	(0.82, 1.14)	0.6789	1.00	(0.84, 1.20)	0.9767
PHGA	1.09	(0.97, 1.22)	0.1518	1.10	(0.97, 1.25)	0.1262
PMA	0.94	(0.83, 1.07)	0.3732	0.97	(0.87, 1.08)	0.5687
NNAL	3.53	(2.43, 5.13)	<.0001	3.52	(2.43, 5.12)	<.0001
NNN	1.08	(0.87, 1.34)	0.4591	1.11	(0.91, 1.36)	0.2860
UCD	1.12	(0.90, 1.40)	0.2987	1.47	(1.27, 1.69)	<.0001
UPB	1.06	(0.87, 1.28)	0.5777	1.28	(1.09, 1.51)	0.0029
UUR	1.11	(0.90, 1.37)	0.3144	1.22	(1.02, 1.48)	0.0332
TNE2	9155	(6503, 12890)	<.0001	8850	(6154, 12729)	<.0001
TNE6	10.27	(5.87, 17.97)	<.0001	8.96	(4.98, 16.10)	<.0001

Source: PATH Study Wave 5 Restricted-Use Files and Biomarker Restricted-Use Files
Note: Sample size varied by BOE. CS: NNAL, N=1338; NNN, N=1309; TNE6, N=1327. DU: NNAL, TNE6, N=114; NNN, N=111. DU+10: NNAL, N=60; NNN, N=58. DU-10: NNN, TNE6, N=53. CE: NNAL, N=149; NNN, N=148; TNE6, N=146. NU: NNAL, N=1828; NNN, N=1836; UCD, UPB, UUR, N=1845; TNE2, N=1842; TNE6, N=152.

Analyses were weighted to represent the US adult civilian, noninstitutionalized population of never, current, and recent (within 1-year) former tobacco users. BOE=Biomarker of Exposure. GMR=Geometric Mean Ratio. 95% CI=95% confidence interval. p-Value=p-value from a t-test of the null hypothesis GMR=1. ENDS=Electronic Nicotine Delivery System. PATH=Population Assessment of Tobacco and Health. Unadjusted GMR=GMR from weighted regression analysis with ln(creatinine-adjusted BOE) as the response variable and tobacco use group as the independent variable. Adjusted GMR=GMR from weighted regression analysis with ln(creatinine-adjusted BOE) as the response variable, tobacco use group as the independent variable, and age, sex, race/ethnicity, and BMI as covariates. GMRs and 95% CI endpoints were computed by exponentiating the estimated contrast and corresponding 95% CI endpoints.

Table S6. Weighted Unadjusted and Adjusted Geometric Mean Ratio by Creatinine-adjusted Biomarker, Two Dual User Strata

Creatinine-adjusted Biomarker	Unadjusted GMR	95% CI	p-Value	Adjusted GMR	95% CI	p-Value
Smokers vs. 10+ CPD Dual Users						
34MH	1.11	(0.68, 1.80)	0.6706	1.17	(0.74, 1.84)	0.5040
AAMA	0.96	(0.75, 1.24)	0.7751	1.02	(0.78, 1.32)	0.8879
AMCA	0.88	(0.68, 1.13)	0.3049	0.95	(0.77, 1.17)	0.6349
CEMA	1.34	(0.65, 2.75)	0.4191	1.27	(0.66, 2.45)	0.4780
CYMA	1.09	(0.56, 2.15)	0.7943	1.13	(0.56, 2.26)	0.7339
HEMA	1.08	(0.52, 2.24)	0.8255	1.19	(0.55, 2.55)	0.6554
HPM2	1.01	(0.61, 1.68)	0.9651	1.14	(0.66, 1.97)	0.6384
HPMA	1.24	(0.56, 2.77)	0.5945	1.24	(0.57, 2.73)	0.5827
HPMM	1.11	(0.60, 2.04)	0.7355	1.12	(0.64, 1.97)	0.6880
IPM3	1.13	(0.56, 2.28)	0.7243	1.21	(0.61, 2.39)	0.5861
MADA	0.85	(0.69, 1.06)	0.1588	0.89	(0.72, 1.09)	0.2448
MHB3	1.03	(0.58, 1.83)	0.9202	1.05	(0.61, 1.83)	0.8537
PHGA	0.91	(0.76, 1.08)	0.2724	0.93	(0.79, 1.10)	0.3763
PMA	0.85	(0.58, 1.24)	0.3959	0.89	(0.61, 1.30)	0.5395
NNAL	0.88	(0.61, 1.25)	0.4611	0.92	(0.65, 1.29)	0.6077
NNN	1.13	(0.54, 2.38)	0.7434	1.22	(0.59, 2.53)	0.5939
UCD	1.24	(0.82, 1.86)	0.3010	1.13	(0.86, 1.49)	0.3889
UPB	1.06	(0.76, 1.50)	0.7158	0.94	(0.73, 1.21)	0.6357
UUR	1.43	(0.79, 2.57)	0.2369	1.52	(0.83, 2.78)	0.1762
TNE2	0.87	(0.57, 1.33)	0.5060	0.95	(0.59, 1.53)	0.8353
TNE6	0.97	(0.59, 1.61)	0.9119	1.00	(0.63, 1.57)	0.9873
Smokers vs. <10 CPD Dual Users						
34MH	2.23	(1.65, 3.03)	<.0001	2.27	(1.67, 3.09)	<.0001
AAMA	1.58	(1.31, 1.91)	<.0001	1.62	(1.33, 1.96)	<.0001
AMCA	1.77	(1.35, 2.33)	<.0001	1.77	(1.38, 2.26)	<.0001
CEMA	1.48	(1.13, 1.95)	0.0055	1.36	(1.03, 1.79)	0.0291
CYMA	4.16	(2.16, 8.04)	<.0001	4.17	(2.14, 8.12)	<.0001
HEMA	1.29	(0.90, 1.85)	0.1623	1.31	(0.90, 1.91)	0.1612
HPM2	1.47	(1.09, 1.96)	0.0108	1.54	(1.11, 2.14)	0.0099
HPMA	2.10	(1.62, 2.72)	<.0001	2.06	(1.57, 2.70)	<.0001
HPMM	2.73	(1.91, 3.89)	<.0001	2.58	(1.76, 3.77)	<.0001
IPM3	3.41	(2.20, 5.28)	<.0001	3.41	(2.16, 5.37)	<.0001
MADA	1.60	(1.37, 1.86)	<.0001	1.57	(1.34, 1.84)	<.0001
MHB3	2.40	(1.73, 3.34)	<.0001	2.33	(1.67, 3.25)	<.0001

PHGA	1.50	(1.25, 1.80)	<.0001	1.49	(1.25, 1.78)	<.0001
PMA	1.17	(0.83, 1.67)	0.3661	1.17	(0.83, 1.65)	0.3781
NNAL	3.85	(2.05, 7.24)	<.0001	3.82	(2.03, 7.17)	<.0001
NNN	2.05	(1.55, 2.71)	<.0001	2.04	(1.51, 2.75)	<.0001
UCD	1.82	(1.42, 2.33)	<.0001	1.36	(1.15, 1.61)	0.0004
UPB	1.40	(1.15, 1.69)	0.0009	1.12	(0.94, 1.34)	0.2130
UUR	0.95	(0.75, 1.21)	0.6905	0.92	(0.73, 1.16)	0.4642
TNE2	1.12	(0.82, 1.53)	0.4837	1.17	(0.86, 1.59)	0.3212
TNE6	1.11	(0.89, 1.39)	0.3395	1.10	(0.88, 1.37)	0.4056
Smokers vs. ENDS Users						
34MH	4.33	(3.46, 5.41)	<.0001	4.26	(3.35, 5.41)	<.0001
AAMA	2.60	(2.26, 3.01)	<.0001	2.61	(2.28, 2.99)	<.0001
AMCA	3.44	(2.97, 3.99)	<.0001	3.27	(2.88, 3.72)	<.0001
CEMA	2.74	(2.27, 3.32)	<.0001	2.58	(2.16, 3.09)	<.0001
CYMA	72.34	(54.94, 95.26)	<.0001	72.54	(55.10, 95.52)	<.0001
HEMA	3.11	(2.54, 3.81)	<.0001	3.08	(2.55, 3.72)	<.0001
HPM2	2.29	(2.03, 2.60)	<.0001	2.32	(2.04, 2.64)	<.0001
HPMA	4.45	(3.70, 5.35)	<.0001	4.38	(3.65, 5.26)	<.0001
HPMM	6.77	(6.04, 7.59)	<.0001	6.40	(5.77, 7.10)	<.0001
IPM3	10.87	(9.05, 13.07)	<.0001	10.66	(8.75, 12.98)	<.0001
MADA	2.03	(1.80, 2.29)	<.0001	1.98	(1.74, 2.25)	<.0001
MHB3	8.12	(6.77, 9.75)	<.0001	7.83	(6.47, 9.47)	<.0001
PHGA	1.75	(1.52, 2.01)	<.0001	1.72	(1.49, 1.99)	<.0001
PMA	1.06	(0.92, 1.22)	0.3911	1.04	(0.92, 1.18)	0.5399
NNAL	86.82	(59.81, 126.01)	<.0001	85.67	(59.09, 124.21)	<.0001
NNN	3.43	(2.81, 4.18)	<.0001	3.35	(2.79, 4.01)	<.0001
UCD	1.80	(1.44, 2.26)	<.0001	1.42	(1.23, 1.64)	<.0001
UPB	1.35	(1.12, 1.63)	0.0017	1.14	(0.99, 1.31)	0.0680
UUR	1.25	(1.02, 1.53)	0.0353	1.17	(0.97, 1.41)	0.1061
TNE2	1.18	(0.85, 1.63)	0.3137	1.21	(0.86, 1.69)	0.2724
TNE6	1.12	(0.93, 1.36)	0.2315	1.10	(0.91, 1.32)	0.3167
Smokers vs. Nonusers						
34MH	5.53	(5.11, 5.98)	<.0001	5.44	(5.06, 5.86)	<.0001
AAMA	2.60	(2.43, 2.78)	<.0001	2.61	(2.44, 2.78)	<.0001
AMCA	4.43	(4.02, 4.89)	<.0001	4.45	(4.02, 4.92)	<.0001
CEMA	3.05	(2.84, 3.28)	<.0001	3.06	(2.86, 3.29)	<.0001
CYMA	123.42	(113.43, 134.29)	<.0001	123.38	(113.09, 134.60)	<.0001
HEMA	3.03	(2.69, 3.42)	<.0001	3.10	(2.75, 3.49)	<.0001

HPM2	2.37	(2.13, 2.64)	<.0001	2.40	(2.15, 2.68)	<.0001
HPMA	5.81	(5.33, 6.33)	<.0001	5.85	(5.35, 6.39)	<.0001
HPMM	5.63	(5.20, 6.10)	<.0001	5.61	(5.19, 6.08)	<.0001
IPM3	12.58	(11.36, 13.93)	<.0001	12.38	(11.21, 13.67)	<.0001
MADA	2.15	(2.03, 2.28)	<.0001	2.16	(2.04, 2.29)	<.0001
MHB3	7.84	(7.33, 8.39)	<.0001	7.84	(7.36, 8.36)	<.0001
PHGA	1.90	(1.76, 2.05)	<.0001	1.89	(1.75, 2.05)	<.0001
PMA	1.00	(0.92, 1.09)	0.9361	1.01	(0.93, 1.09)	0.8439
NNAL	306.44	(274.65, 341.89)	<.0001	301.49	(269.99, 336.67)	<.0001
NNN	3.72	(3.33, 4.14)	<.0001	3.72	(3.35, 4.14)	<.0001
UCD	2.02	(1.87, 2.19)	<.0001	2.08	(1.95, 2.23)	<.0001
UPB	1.43	(1.31, 1.55)	<.0001	1.46	(1.35, 1.59)	<.0001
UUR	1.39	(1.24, 1.55)	<.0001	1.43	(1.27, 1.60)	<.0001
TNE2	10807	(9206, 12687)	<.0001	10681	(9065, 12584)	<.0001
TNE6	11.52	(7.02, 18.90)	<.0001	9.83	(5.81, 16.66)	<.0001
10+ CPD Dual Users vs. <10 CPD Dual Users						
34MH	2.01	(1.11, 3.64)	0.0209	1.95	(1.10, 3.43)	0.0219
AAMA	1.64	(1.19, 2.25)	0.0027	1.59	(1.14, 2.21)	0.0068
AMCA	2.02	(1.39, 2.95)	0.0003	1.86	(1.33, 2.58)	0.0003
CEMA	1.10	(0.51, 2.38)	0.7988	1.07	(0.52, 2.19)	0.8474
CYMA	3.81	(1.44, 10.05)	0.0074	3.70	(1.38, 9.91)	0.0099
HEMA	1.19	(0.53, 2.70)	0.6718	1.10	(0.47, 2.59)	0.8223
HPM2	1.45	(0.80, 2.64)	0.2227	1.35	(0.70, 2.62)	0.3669
HPMA	1.70	(0.72, 3.98)	0.2224	1.65	(0.71, 3.84)	0.2396
HPMM	2.46	(1.19, 5.05)	0.0151	2.30	(1.15, 4.60)	0.0191
IPM3	3.01	(1.30, 6.96)	0.0106	2.82	(1.23, 6.48)	0.0150
MADA	1.87	(1.43, 2.44)	<.0001	1.77	(1.37, 2.30)	<.0001
MHB3	2.33	(1.19, 4.57)	0.0140	2.21	(1.16, 4.23)	0.0170
PHGA	1.65	(1.29, 2.12)	0.0001	1.60	(1.27, 2.03)	0.0001
PMA	1.38	(0.84, 2.26)	0.1961	1.31	(0.80, 2.15)	0.2768
NNAL	4.40	(2.07, 9.33)	0.0002	4.17	(1.99, 8.75)	0.0002
NNN	1.81	(0.81, 4.05)	0.1458	1.68	(0.76, 3.69)	0.1973
UCD	1.47	(0.93, 2.32)	0.0957	1.21	(0.88, 1.66)	0.2384
UPB	1.31	(0.88, 1.95)	0.1770	1.19	(0.87, 1.62)	0.2742
UUR	0.67	(0.35, 1.26)	0.2101	0.61	(0.32, 1.15)	0.1253
TNE2	1.29	(0.77, 2.17)	0.3318	1.23	(0.72, 2.11)	0.4506
TNE6	1.15	(0.66, 1.98)	0.6213	1.10	(0.66, 1.83)	0.7087
10+ CPD Dual Users vs. ENDS Users						

34MH	3.90	(2.28, 6.68)	<.0001	3.65	(2.18, 6.10)	<.0001
AAMA	2.70	(2.07, 3.53)	<.0001	2.56	(1.94, 3.39)	<.0001
AMCA	3.93	(2.96, 5.22)	<.0001	3.44	(2.72, 4.36)	<.0001
CEMA	2.04	(0.98, 4.28)	0.0580	2.04	(1.03, 4.04)	0.0417
CYMA	66.17	(32.15, 136.20)	<.0001	64.36	(30.95, 133.79)	<.0001
HEMA	2.87	(1.35, 6.09)	0.0066	2.59	(1.18, 5.69)	0.0183
HPM2	2.27	(1.35, 3.80)	0.0022	2.04	(1.15, 3.59)	0.0147
HPMA	3.58	(1.58, 8.11)	0.0025	3.52	(1.58, 7.85)	0.0024
HPMM	6.10	(3.30, 11.29)	<.0001	5.71	(3.23, 10.08)	<.0001
IPM3	9.60	(4.67, 19.73)	<.0001	8.83	(4.35, 17.92)	<.0001
MADA	2.38	(1.87, 3.03)	<.0001	2.23	(1.78, 2.79)	<.0001
MHB3	7.89	(4.30, 14.47)	<.0001	7.43	(4.15, 13.32)	<.0001
PHGA	1.93	(1.57, 2.37)	<.0001	1.85	(1.52, 2.25)	<.0001
PMA	1.25	(0.85, 1.83)	0.2526	1.17	(0.79, 1.73)	0.4313
NNAL	99.10	(60.64, 161.95)	<.0001	93.62	(58.54, 149.72)	<.0001
NNN	3.03	(1.40, 6.55)	0.0052	2.75	(1.30, 5.82)	0.0087
UCD	1.46	(0.93, 2.28)	0.0961	1.26	(0.92, 1.72)	0.1445
UPB	1.27	(0.86, 1.88)	0.2305	1.21	(0.90, 1.63)	0.2029
UUR	0.88	(0.47, 1.63)	0.6723	0.77	(0.41, 1.44)	0.4081
TNE2	1.36	(0.80, 2.33)	0.2568	1.27	(0.72, 2.24)	0.4077
TNE6	1.15	(0.68, 1.97)	0.5978	1.10	(0.68, 1.80)	0.6942
10+ CPD Dual Users vs. Nonusers						
34MH	4.98	(3.07, 8.10)	<.0001	4.66	(2.95, 7.38)	<.0001
AAMA	2.69	(2.09, 3.46)	<.0001	2.56	(1.96, 3.33)	<.0001
AMCA	5.06	(3.89, 6.58)	<.0001	4.67	(3.73, 5.86)	<.0001
CEMA	2.27	(1.10, 4.70)	0.0269	2.42	(1.24, 4.70)	0.0098
CYMA	112.90	(57.33, 222.34)	<.0001	109.46	(54.36, 220.41)	<.0001
HEMA	2.79	(1.34, 5.81)	0.0064	2.61	(1.20, 5.64)	0.0156
HPM2	2.34	(1.39, 3.94)	0.0016	2.11	(1.20, 3.68)	0.0096
HPMA	4.68	(2.09, 10.48)	0.0003	4.70	(2.12, 10.40)	0.0002
HPMM	5.08	(2.75, 9.38)	<.0001	5.01	(2.83, 8.85)	<.0001
IPM3	11.10	(5.45, 22.62)	<.0001	10.25	(5.11, 20.56)	<.0001
MADA	2.52	(2.03, 3.13)	<.0001	2.44	(1.99, 2.99)	<.0001
MHB3	7.62	(4.26, 13.62)	<.0001	7.45	(4.27, 13.00)	<.0001
PHGA	2.09	(1.76, 2.49)	<.0001	2.04	(1.71, 2.43)	<.0001
PMA	1.18	(0.81, 1.72)	0.3857	1.13	(0.78, 1.64)	0.5032
NNAL	349.79	(243.69, 502.10)	<.0001	329.47	(232.11, 467.69)	<.0001
NNN	3.29	(1.56, 6.91)	0.0020	3.06	(1.47, 6.35)	0.0031
UCD	1.64	(1.08, 2.47)	0.0196	1.85	(1.39, 2.45)	<.0001

UPB	1.34	(0.96, 1.88)	0.0893	1.56	(1.20, 2.01)	0.0009
UUR	0.97	(0.54, 1.76)	0.9279	0.94	(0.51, 1.73)	0.8459
TNE2	12476	(7975, 19518)	<.0001	11232	(6747, 18700)	<.0001
TNE6	11.85	(5.91, 23.76)	<.0001	9.87	(4.90, 19.89)	<.0001
<10 CPD Dual Users vs. ENDS Users						
34MH	1.94	(1.31, 2.85)	0.0010	1.87	(1.26, 2.79)	0.0022
AAMA	1.65	(1.25, 2.17)	0.0005	1.62	(1.24, 2.12)	0.0006
AMCA	1.94	(1.40, 2.69)	0.0001	1.85	(1.39, 2.48)	<.0001
CEMA	1.85	(1.36, 2.53)	0.0002	1.90	(1.41, 2.57)	<.0001
CYMA	17.37	(8.01, 37.66)	<.0001	17.41	(8.01, 37.85)	<.0001
HEMA	2.41	(1.59, 3.64)	<.0001	2.35	(1.55, 3.55)	<.0001
HPM2	1.56	(1.13, 2.16)	0.0070	1.50	(1.07, 2.12)	0.0194
HPMA	2.11	(1.58, 2.83)	<.0001	2.13	(1.58, 2.86)	<.0001
HPMM	2.48	(1.68, 3.68)	<.0001	2.48	(1.65, 3.73)	<.0001
IPM3	3.19	(1.97, 5.17)	<.0001	3.13	(1.89, 5.18)	<.0001
MADA	1.27	(1.03, 1.57)	0.0255	1.26	(1.02, 1.55)	0.0316
MHB3	3.38	(2.34, 4.88)	<.0001	3.36	(2.32, 4.86)	<.0001
PHGA	1.17	(0.92, 1.48)	0.2078	1.15	(0.92, 1.45)	0.2236
PMA	0.90	(0.63, 1.31)	0.5877	0.89	(0.63, 1.27)	0.5178
NNAL	22.52	(10.47, 48.46)	<.0001	22.45	(10.48, 48.07)	<.0001
NNN	1.67	(1.21, 2.32)	0.0023	1.64	(1.20, 2.24)	0.0022
UCD	0.99	(0.72, 1.37)	0.9576	1.04	(0.83, 1.30)	0.7164
UPB	0.97	(0.75, 1.25)	0.8054	1.02	(0.81, 1.28)	0.8701
UUR	1.31	(0.94, 1.81)	0.1043	1.27	(0.93, 1.73)	0.1289
TNE2	1.06	(0.67, 1.67)	0.8123	1.03	(0.65, 1.64)	0.8902
TNE6	1.01	(0.75, 1.35)	0.9632	1.00	(0.77, 1.30)	0.9937
<10 CPD Dual Users vs. Nonusers						
34MH	2.47	(1.81, 3.38)	<.0001	2.40	(1.76, 3.27)	<.0001
AAMA	1.64	(1.35, 2.01)	<.0001	1.61	(1.32, 1.97)	<.0001
AMCA	2.50	(1.85, 3.38)	<.0001	2.52	(1.89, 3.35)	<.0001
CEMA	2.06	(1.55, 2.74)	<.0001	2.26	(1.70, 2.99)	<.0001
CYMA	29.64	(15.45, 56.85)	<.0001	29.61	(15.29, 57.36)	<.0001
HEMA	2.34	(1.63, 3.38)	<.0001	2.37	(1.61, 3.48)	<.0001
HPM2	1.62	(1.21, 2.16)	0.0014	1.56	(1.13, 2.15)	0.0080
HPMA	2.76	(2.14, 3.57)	<.0001	2.84	(2.17, 3.72)	<.0001
HPMM	2.07	(1.44, 2.97)	0.0001	2.18	(1.48, 3.21)	0.0001
IPM3	3.69	(2.36, 5.78)	<.0001	3.63	(2.28, 5.80)	<.0001
MADA	1.35	(1.17, 1.56)	<.0001	1.37	(1.18, 1.60)	<.0001

MHB3	3.26	(2.33, 4.57)	<.0001	3.37	(2.40, 4.73)	<.0001
PHGA	1.27	(1.05, 1.52)	0.0127	1.27	(1.06, 1.53)	0.0107
PMA	0.85	(0.61, 1.20)	0.3638	0.86	(0.62, 1.21)	0.3899
NNAL	79.50	(40.84, 154.76)	<.0001	78.99	(40.60, 153.68)	<.0001
NNN	1.81	(1.34, 2.45)	0.0002	1.82	(1.33, 2.51)	0.0003
UCD	1.11	(0.86, 1.44)	0.4114	1.53	(1.29, 1.81)	<.0001
UPB	1.02	(0.85, 1.24)	0.8137	1.31	(1.08, 1.58)	0.0056
UUR	1.46	(1.16, 1.83)	0.0017	1.56	(1.24, 1.95)	0.0002
TNE2	9671	(6805, 13745)	<.0001	9140	(6392, 13070)	<.0001
TNE6	10.34	(6.11, 17.49)	<.0001	8.97	(5.16, 15.57)	<.0001
ENDS Users vs. Nonusers						
34MH	1.28	(1.03, 1.58)	0.0271	1.28	(1.01, 1.61)	0.0378
AAMA	1.00	(0.86, 1.16)	0.9737	1.00	(0.86, 1.15)	0.9684
AMCA	1.29	(1.10, 1.51)	0.0018	1.36	(1.17, 1.57)	<.0001
CEMA	1.11	(0.92, 1.34)	0.2636	1.19	(0.99, 1.42)	0.0586
CYMA	1.71	(1.32, 2.20)	<.0001	1.70	(1.32, 2.18)	<.0001
HEMA	0.97	(0.80, 1.18)	0.7912	1.01	(0.84, 1.21)	0.9461
HPM2	1.03	(0.89, 1.19)	0.6568	1.03	(0.89, 1.21)	0.6667
HPMA	1.31	(1.10, 1.56)	0.0033	1.33	(1.12, 1.59)	0.0016
HPMM	0.83	(0.74, 0.94)	0.0028	0.88	(0.78, 0.98)	0.0220
IPM3	1.16	(0.99, 1.36)	0.0745	1.16	(0.96, 1.40)	0.1135
MADA	1.06	(0.95, 1.18)	0.2861	1.09	(0.97, 1.23)	0.1441
MHB3	0.97	(0.82, 1.14)	0.6789	1.00	(0.84, 1.20)	0.9812
PHGA	1.09	(0.97, 1.22)	0.1518	1.10	(0.97, 1.25)	0.1267
PMA	0.94	(0.83, 1.07)	0.3732	0.97	(0.87, 1.08)	0.5666
NNAL	3.53	(2.43, 5.13)	<.0001	3.52	(2.42, 5.11)	<.0001
NNN	1.08	(0.87, 1.34)	0.4591	1.11	(0.91, 1.36)	0.2874
UCD	1.12	(0.90, 1.40)	0.2987	1.47	(1.27, 1.69)	<.0001
UPB	1.06	(0.87, 1.28)	0.5777	1.28	(1.09, 1.51)	0.0029
UUR	1.11	(0.90, 1.37)	0.3144	1.23	(1.02, 1.48)	0.0327
TNE2	9155	(6503, 12890)	<.0001	8849	(6153, 12726)	<.0001
TNE6	10.27	(5.87, 17.97)	<.0001	8.96	(4.98, 16.09)	<.0001

Source: PATH Study Wave 5 Restricted-Use Files and Biomarker Restricted-Use Files

Note: Sample size varied by BOE. CS: NNAL, N=1338; NNN, N=1309; TNE6, N=1327. DU: NNAL, TNE6, N=114; NNN, N=111. DU+10: NNAL, N=60; NNN, N=58. DU-10: NNN, TNE6, N=53. CE: NNAL, N=149; NNN, N=148; TNE6, N=146. NU: NNAL, N=1828; NNN, N=1836; UCD, UPB, UUR, N=1845; TNE2, N=1842; TNE6, N=152.

Analyses were weighted to represent the US adult civilian, noninstitutionalized population of never, current, and recent (within 1-year) former tobacco users. BOE=Biomarker of Exposure. GMR=Geometric Mean Ratio. 95% CI=95% confidence interval. p-Value=p-value from a t-test of the null hypothesis GMR=1. ENDS=Electronic Nicotine Delivery System. PATH=Population Assessment of Tobacco and Health. Unadjusted GMR=GMR from weighted regression analysis with ln(creatinine-adjusted BOE) as the response variable and tobacco use group as the

independent variable. Adjusted GMR=GMR from weighted regression analysis with ln(creatinine-adjusted BOE) as the response variable, tobacco use group as the independent variable, and age, sex, race/ethnicity, and BMI as covariates. GMRs and 95% CI endpoints were computed by exponentiating the estimated contrast and corresponding 95% CI endpoints.

Figure S1. Nicotine Equivalents among Smokers, ENDS Users, Dual Users and Tobacco Nonusers (Weighted Adjusted Geometric Mean and 95% Confidence Interval)

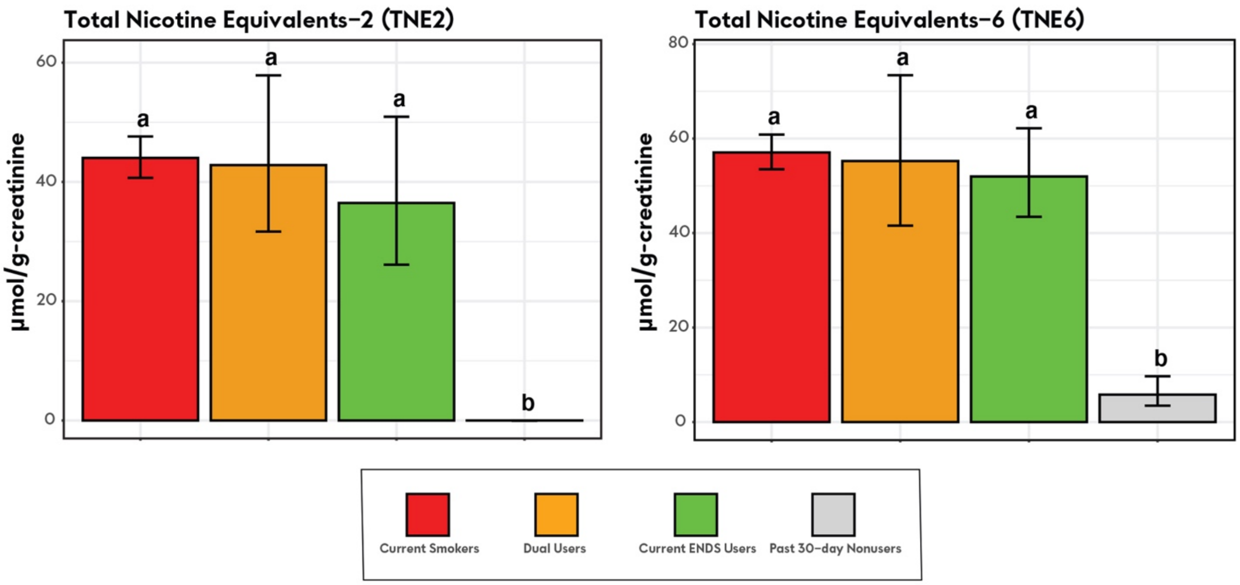

Source: PATH Study Wave 5 Restricted-Use Files and Biomarker Restricted-Use Files
Note. Groups whose bars do not share a letter above the bar are significantly different ($p < 0.05$). Groups whose bars share a letter do not significantly differ from each other ($p \geq 0.05$). Adjusted geometric means and confidence interval endpoints were derived from a weighted regression analysis with covariates for age, sex, race/ethnicity, and BMI and were computed by exponentiating predicted population margins with covariate values fixed at the observed margins. Analyses were weighted to represent the US adult civilian, noninstitutionalized population of never, current, and recent (within 1-year) former tobacco users. Current Smokers: TNE2, N=1341; TNE6 N=1327. Dual Users: TNE6, N=114. Current ENDS Users: TNE2, N=151; TNE6, N=146. Past 30-day Nonusers: TNE2, N=1842; TNE6 N=152.

Figure S2. BOEs of Tobacco-Specific Nitrosamines among Smokers, ENDS Users, Dual Users and Past 30-day Nonusers (Weighted Adjusted Geometric Mean and 95% Confidence Interval)

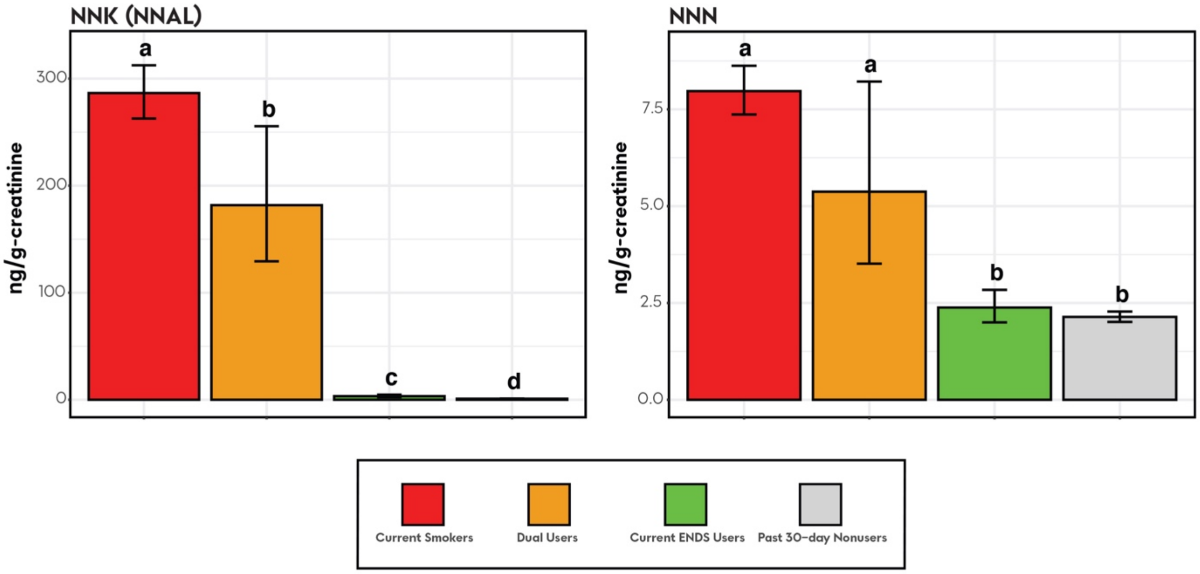

Source: PATH Study Wave 5 Restricted-Use Files and Biomarker Restricted-Use Files
Note. Groups whose bars do not share a letter above the bar are significantly different ($p < 0.05$). Groups whose bars share a letter do not significantly differ from each other ($p \geq 0.05$). Adjusted geometric means and confidence interval endpoints were derived from a weighted regression analysis with covariates for age, sex, race/ethnicity, and BMI and were computed by exponentiating predicted population margins with covariate values fixed at the observed margins. Analyses were weighted to represent the US adult civilian, noninstitutionalized population of never, current, and recent (within 1-year) former tobacco users. Current Smokers: NNAL, N=1338; NNN, N=1309. Dual Users: NNAL, N=114; NNN, N=111. Current ENDS Users: NNAL, N=149; NNN, N=148. Past 30-day Nonusers: NNAL, N=1828; NNN, N=1836.

Figure S3. BOEs of VOCs among Smokers, ENDS Users, Dual Users and Past 30-day Nonusers (Weighted Adjusted Geometric Mean and 95% Confidence Interval)

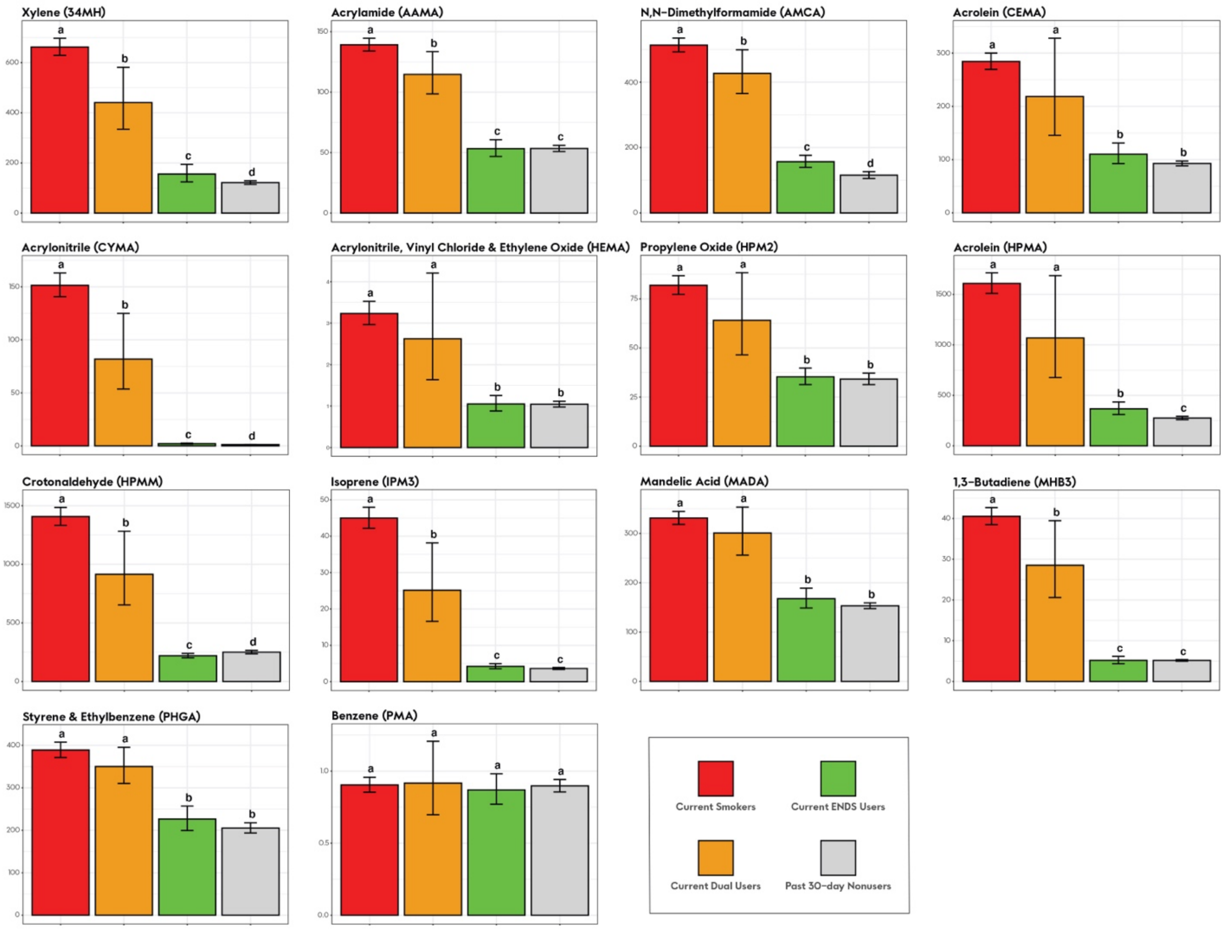

Source: PATH Study Wave 5 Restricted-Use Files and Biomarker Restricted-Use Files

Note. All values represent µg/g creatinine. Groups whose bars do not share a letter above the bar are significantly different ($p < 0.05$). Groups whose bars share a letter do not significantly differ from each other ($p \geq 0.05$). Adjusted geometric means and confidence interval endpoints were derived from a weighted regression analysis with covariates for age, sex, race/ethnicity, and BMI and were computed by exponentiating predicted population margins with covariate values fixed at the observed margins.

Analyses were weighted to represent the US adult civilian, noninstitutionalized population of never, current, and recent (within 1-year) former tobacco users. Current Smokers, N=1341; Dual Users, N=115; Current ENDS Users, N=151; Past 30-day Nonusers, N=1846.

Figure S4. BOEs of Metals among Smokers, ENDS Users, Dual Users and Past 30-day Nonusers (Weighted Adjusted Geometric Mean and 95% Confidence Interval)

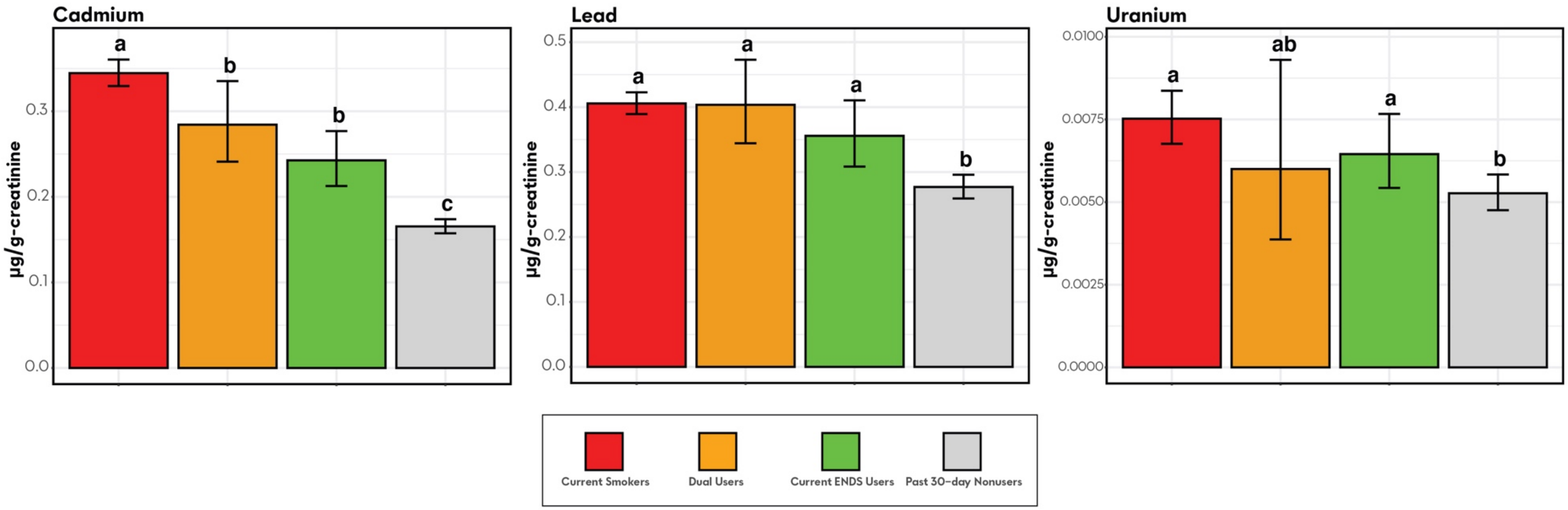

Source: PATH Study Wave 5 Restricted-Use Files and Biomarker Restricted-Use Files
Note. Groups whose bars do not share a letter above the bar are significantly different ($p < 0.05$). Groups whose bars share a letter do not significantly differ from each other ($p \geq 0.05$). Adjusted geometric means and confidence interval endpoints were derived from a weighted regression analysis with covariates for age, sex, race/ethnicity, and BMI and were computed by exponentiating predicted population margins with covariate values fixed at the observed margins. Analyses were weighted to represent the US adult civilian, noninstitutionalized population of never, current, and recent (within 1-year) former tobacco users. Current Smokers, N=1341; Dual Users, N=115; Current ENDS Users, N=151; Past 30-day Nonusers, N=1845.
